# Economic evaluation of the NET intervention versus guideline dissemination for management of mild head injury in hospital emergency departments

**DOI:** 10.1186/s13012-018-0834-6

**Published:** 2018-12-05

**Authors:** Duncan Mortimer, Marije Bosch, Joanne E. Mckenzie, Simon Turner, Marisa Chau, Jennie L. Ponsford, Jonathan C. Knott, Russell L. Gruen, Sally E. Green

**Affiliations:** 1 Centre for Health Economics, Monash Business School, Monash University, Melbourne, Australia; 2 Department of Surgery, Monash University, Melbourne, Australia; 3 National Trauma Research Institute, Alfred Hospital and Monash University, Melbourne, Australia; 4 Faculty of Economics and Business, University of Groningen, Groningen, The Netherlands; 5 School of Public Health and Preventive Medicine, Monash University, Melbourne, Australia; 6 Melbourne Medical School, The University of Melbourne, Melbourne, Australia; 7 Department of Emergency Medicine, Royal Melbourne Hospital, Melbourne, Australia; 8 Monash-Epworth Rehabilitation Research Centre, Epworth Hospital, Melbourne, Australia; 9 School of Psychological Sciences, Monash University, Melbourne, Australia; 10 Lee Kong Chian School of Medicine, Nanyang Technological University, Melbourne, Singapore

**Keywords:** Mild head injury, Mild traumatic brain injury, Emergency medicine, Implementation science, Clinical practice guideline, Evidence-based practice, Cost-effectiveness

## Abstract

**Background:**

Evidence-based guidelines for the management of mild traumatic brain injury (mTBI) in the emergency department (ED) are now widely available, and yet, clinical practice remains inconsistent with the guidelines. The Neurotrauma Evidence Translation (NET) intervention was developed to increase the uptake of guideline recommendations and improve the management of minor head injury in Australian emergency departments (EDs). However, the adoption of this type of intervention typically entails an upfront investment that may or may not be fully offset by improvements in clinical practice, health outcomes and/or reductions in health service utilisation. The present study estimates the cost and cost-effectiveness of the NET intervention, as compared to the passive dissemination of the guideline, to evaluate whether any improvements in clinical practice or health outcomes due to the NET intervention can be obtained at an acceptable cost.

**Methods and findings:**

Study setting: The NET cluster randomised controlled trial [ACTRN12612001286831]. Study sample: Seventeen EDs were randomised to the control condition and 14 to the intervention. One thousand nine hundred forty-three patients were included in the analysis of clinical practice outcomes (NET sample). A total of 343 patients from 14 control and 10 intervention EDs participated in follow-up interviews and were included in the analysis of patient-reported health outcomes (NET-Plus sample). Outcome measures: Appropriate post-traumatic amnesia (PTA) screening in the ED (primary outcome). Secondary clinical practice outcomes: provision of written information on discharge (INFO) and safe discharge (defined as CT scan appropriately provided plus PTA plus INFO). Secondary patient-reported, post-discharge health outcomes: anxiety (Hospital Anxiety and Depression Scale), post-concussive symptoms (Rivermead), and preference-based health-related quality of life (SF6D). Methods: Trial-based economic evaluations from a health sector perspective, with time horizons set to coincide with the final follow-up for the NET sample (2 months post-intervention) and to 1-month post-discharge for the NET-Plus sample. Results: Intervention and control groups were not significantly different in health service utilisation received in the ED/inpatient ward following the initial mTBI presentation (adjusted mean difference $23.86 per patient; 95%CI − $106, $153; p = 0.719) or over the longer follow-up in the NET-plus sample (adjusted mean difference $341.78 per patient; 95%CI − $58, $742; p = 0.094). Savings from lower health service utilisation are therefore unlikely to offset the significantly higher upfront cost of the intervention (mean difference $138.20 per patient; 95%CI $135, $141; *p* < 0.000). Estimates of the net effect of the intervention on total cost (intervention cost net of health service utilisation) suggest that the intervention entails significantly higher costs than the control condition (adjusted mean difference $169.89 per patient; 95%CI $43, $297, p = 0.009). This effect is larger in absolute magnitude over the longer follow-up in the NET-plus sample (adjusted mean difference $505.06; 95%CI $96, $915; p = 0.016), mostly due to additional health service utilisation. For the primary outcome, the NET intervention is more costly and more effective than passive dissemination; entailing an additional cost of $1246 per additional patient appropriately screened for PTA ($169.89/0.1363; Fieller’s 95%CI $525, $2055). For NET to be considered cost-effective with 95% confidence, decision-makers would need to be willing to trade one quality-adjusted life year (QALY) for 25 additional patients appropriately screened for PTA. While these results reflect our best estimate of cost-effectiveness given the data, it is possible that a NET intervention that has been scaled and streamlined ready for wider roll-out may be more or less cost-effective than the NET intervention as delivered in the trial.

**Conclusions:**

While the NET intervention does improve the management of mTBI in the ED, it also entails a significant increase in cost and—as delivered in the trial—is unlikely to be cost-effective at currently accepted funding thresholds. There may be a scope for a scaled-up and streamlined NET intervention to achieve a better balance between costs and outcomes.

**Trial registration:**

Australian New Zealand Clinical Trials Registry ACTRN12612001286831, date registered 12 December 2012.

**Electronic supplementary material:**

The online version of this article (10.1186/s13012-018-0834-6) contains supplementary material, which is available to authorized users.

## Introduction

Evidence-based guidelines for the management of mTBI in the ED are now widely available [1], and yet, clinical practice remains inconsistent with key guideline recommendations (see [2], Table 1). A number of previous studies have estimated the costs and benefits associated with adherence to alternative management strategies for mTBI including selective CT scanning, CT for all patients, skull radiography for all patients, prolonged ED observation, 24-h hospital admission, and no treatment [3–5]. Findings suggest that—at the mean—selective CT scanning is cost-effective in comparison to other strategies and that alternative criteria for selective CT offer ‘broadly similar costs and quality-adjusted life years’ [3] (page 1428). However, each of these previous studies evaluated the cost-effectiveness of perfect adherence to the evaluated management strategies [6]; ignoring the fact that achieving perfect adherence is a difficult and costly undertaking [7–9]. Moreover, each is primarily concerned with adherence to *diagnostic* management strategies. Evidence-based guidelines for the management of mTBI in the ED include recommendations that extend beyond diagnostic management such as provision of verbal and written patient information upon discharge, and evidence-practice gaps are equally problematic for these recommendations (see [2], Table 1).

**Table 1:** Schedule of measures for the economic evaluation

Measures	Data collection	Timing	Source	Level
Clinical practice outcomes				
Appropriate PTA screening (PTA)^1^	Chart audit	Retrospectively for 2 months period post-intervention	ED medical records of eligible patients	Patient
Provision of written patient information (INFO)				
Safe discharge (SAFED)^2^				
Clinical patient outcomes and health-related quality of life				
Anxiety^3^	Telephone interview	Ave. 210 days post discharge (SD 38.5 days; IQR 181–239; min = 130, max = 321)	Patient self-report	Patient
Post-concussive symptoms^4^				
HRQoL^5^				
Direct cost of the intervention				
Preparation/delivery time and attendance for local training sessions	Questionnaire	On completion of delivery to all EDs	Clinician self-report	ED
Direct cost of all other intervention components	Data abstraction and interview	On completion of delivery to all EDs	Administrative and financial records	ED
Health care utilisation and costs				
Medical and surgical services received in the ED/inpatient ward (including CT scan)	Chart audit	Retrospectively on 2 months period post-intervention	ED medical records of eligible patients	Patient
Re-presentation to ED within 1 month of mTBI				
Post-discharge mTBI-related service utilisation	Telephone interview	Ave. 210 days post discharge (SD 38.5 days; IQR 181–239; min = 130, max = 321)	Patient self-report	Patient

^1^Primary outcome for the economic evaluation

^2^Defined as PTA, INFO, and CT where CT denotes whether a CT scan was provided in the presence of a risk factor that justifies the scan (age 65 or older; GCS < 15; amnesia; suspected skull fracture; vomiting and coagulopathy) [26] (assessed in the cohort of patients for whom risk criteria were recorded only). CT therefore indicates whether a scan was appropriately provided but not whether a scan was ‘appropriately denied’. CT and SAFED only assessed in the cohort of patients for whom risk criteria were recorded

^3^Anxiety measured using the relevant questions in the Hospital Anxiety and Depression Scale giving a score between 0 and 21, higher scores indicate higher levels of anxiety, and a score > 7 indicative of clinically significant anxiety

^4^Post-concussion symptoms measured using the 13-item Rivermead scale giving a score between 0 and 52, higher scores indicate greater severity of post-concussion symptoms

^5^12-item short form health survey (SF-12 v2) to derive SF-12-based SF6D index scores using the UK weights from Brazier and Roberts [20]. SF12-based SF6D index scores range between 0.350 (the ‘pits’) and 1.000 (full health)

The Neurotrauma Evidence Translation (NET) intervention was developed to increase the uptake of three key recommendations for the management of adult patients who present to Australian EDs with mTBI [10]. The three recommendations targeted by the NET intervention are as follows:

- Post-traumatic amnesia (PTA) should be prospectively assessed in the emergency department using a validated tool.
- Guideline-developed criteria or clinical decision rules should be used to determine the appropriate use and timing of computed tomography (CT) imaging.
- Verbal and written patient information (consisting of advice, education, and reassurance) should be provided upon discharge from the emergency department (INFO).

The development of the NET intervention was informed by empirical evidence and two theoretical frameworks, selected for their relevance to changing clinician behaviour via implementation interventions in a complex organisational environment [10]. Specifically, factors influencing clinical behaviour change were identified from the Theoretical Domains Framework [11]; organisational factors likely to influence the uptake and implementation of interventions in complex environments such as EDs were identified from the Model of Diffusion of Innovations in Service Organisations [12]. This approach yielded an intervention designed to operate at the level of the clinician and the organisation and to target factors hypothesised to influence uptake of practice recommendations in EDs [13, 14].

The NET Trial [2] aimed to test the effectiveness of the NET intervention for improving management of mTBI in Australian EDs, as compared with the passive dissemination of the relevant guideline [15]. The NET trial estimated the effects of the NET intervention on clinical practice outcomes (NET sample) and on patient-reported health outcomes in a smaller sample of EDs and patients who participated in follow-up interviews (NET-Plus sample). Results from the NET Trial suggest that the NET intervention improves the management of mTBI in the ED; increasing a patient’s chances of being appropriately assessed for PTA and of ‘safe discharge’ (Bosch M, McKenzie J, Ponsford JL, Turner S, Chau M, Tavender EJ, et al.: Evaluation of a targeted, theory-informed implementation intervention designed to increase uptake of emergency management recommendations regarding adult patients with mild traumatic brain injury: results of the NET cluster randomised controlled trial, Forthcoming). However, the improvement in PTA did not clear an a priori specified threshold for clinical significance and the improvement in ‘safe discharge’ mainly reflects the improvement in PTA [2], (Bosch M, McKenzie J, Ponsford JL, Turner S, Chau M, Tavender EJ, et al.: Evaluation of a targeted, theory-informed implementation intervention designed to increase uptake of emergency management recommendations regarding adult patients with mild traumatic brain injury: results of the NET cluster randomised controlled trial, Forthcoming). Detailed results from the main effectiveness analysis of the NET Trial are reported elsewhere (Bosch M, McKenzie J, Ponsford JL, Turner S, Chau M, Tavender EJ, et al.: Evaluation of a targeted, theory-informed implementation intervention designed to increase uptake of emergency management recommendations regarding adult patients with mild traumatic brain injury: results of the NET cluster randomised controlled trial, Forthcoming).

(Bosch M, McKenzie J, Ponsford JL, Turner S, Chau M, Tavender EJ, et al.: Evaluation of a targeted, theory-informed implementation intervention designed to increase uptake of emergency management recommendations regarding adult patients with mild traumatic brain injury: results of the NET cluster randomised controlled trial, Forthcoming) argued that understanding the impact of the intervention on health service utilisation and total cost may be necessary before clear policy recommendations can be drawn from the NET trial. For example, the NET intervention has the potential to reduce some categories of health service utilisation including diagnostic imaging in the ED and unplanned hospital readmissions. The NET intervention also entails an upfront investment that may or may not be fully offset by improvements in clinical practice, health outcomes, and/or reductions in health service utilisation. The economic evaluation described here will explicitly consider this trade-off; first estimating the impact of the intervention on health service utilisation and total cost and then combining treatment effects with respect to cost (incremental cost) and effectiveness (incremental effectiveness) to quantify the incremental cost-effectiveness (incremental cost divided by incremental effectiveness) of the NET intervention.

The complexity of this trade-off has a broader relevance for evidence-based medicine. Closing gaps between evidence and practice is rarely a simple matter [16, 17], and changing clinician behaviour has proven difficult even with tailored, multi-faceted interventions [18, 19]. On the cost side of the equation, attempts to maximise effectiveness by adding facets and complexity, or via the use of tailored rather than ‘off-the-shelf’ interventions, will typically add cost and this may or may not be money well-spent [8, 20]. The present study demonstrates the importance and value of quantifying trade-offs between cost and effectiveness and serves as a reminder that initiatives to increase adherence to guideline recommendations should themselves offer good value for money.

## Methods

### Study design

The NET study [ACTRN12612001286831] was a cluster randomised controlled trial (CRT) designed to test the effectiveness of the NET intervention for improving management of mTBI in Australian EDs, as compared with the passive dissemination of the relevant guideline [15]. For the present study, trial-based economic evaluations were conducted alongside the NET CRT to quantify the incremental cost-effectiveness of NET intervention in achieving practice change and health outcomes, as compared to the dissemination of the relevant clinical practice guideline. All analyses were conducted from a health sector perspective. The time horizons for the inclusion of relevant costs and consequences coincide with the final scheduled follow-up for the NET sample (2 months post-intervention) and to 1 month post-discharge for the NET-Plus sample. Ethical approval for this study was initially obtained from the Alfred Health Human Research Ethics Committee (approval Number 398/12); local ethics and research governance procedures were subsequently completed for each study site. Please refer to our study protocol [2] for a detailed description of the design of the CRT and methods for the accompanying trial-based economic evaluations.

### Study setting and study sample

The economic evaluations alongside the NET study made use of the data at the ED, clinician and patient levels; with recruitment and randomisation procedures for EDs, clinicians, and patients as described in the study protocol [2] and main trial report (Bosch M, McKenzie J, Ponsford JL, Turner S, Chau M, Tavender EJ, et al.: Evaluation of a targeted, theory-informed implementation intervention designed to increase uptake of emergency management recommendations regarding adult patients with mild traumatic brain injury: results of the NET cluster randomised controlled trial, Forthcoming). Briefly, 24-h Australian EDs meeting inclusion criteria whose Directors consented to participate in either NET or NET-Plus were allocated to either the intervention or control group using the method of minimisation. EDs operating for less than 24 h per day, EDs without a CT scanner on site, and EDs in specialised hospitals that do not routinely treat adult patients with TBI (for example, women’s, or children) were excluded. Based on estimates of required sample size (15 EDs per group), to detect a clinically significant 20% change in the primary outcome at the 5% level with approximately 80% power, we aimed to recruit 34 EDs to allow for 10% attrition.

For included EDs, a sample of mTBI patients presenting in the 2 months directly following the local intervention delivery period was selected for inclusion in chart audit using the three-step process described by (Bosch M, McKenzie J, Ponsford JL, Turner S, Chau M, Tavender EJ, et al.: Evaluation of a targeted, theory-informed implementation intervention designed to increase uptake of emergency management recommendations regarding adult patients with mild traumatic brain injury: results of the NET cluster randomised controlled trial, Forthcoming). Patients were eligible for inclusion if they were aged 18 or older, had presented to the ED within 24 h of injury, sustained an acute blunt head trauma, and had a Glasgow Coma Scale score of 14 or 15 at presentation. Patients with penetrating injuries or non-traumatic brain-injury such as stroke were excluded. Patients included in the chart audit and drawn from NET-Plus EDs were subsequently invited to participate in the NET-Plus follow-up of patient-reported health outcomes. At the clinician level, a sample of 50 medical and 50 nursing staff was randomly selected for inclusion in the data collection from a broader list of medical and nursing staff meeting inclusion/exclusion criteria in each participating ED.

### Intervention and comparator

EDs randomised to the control group received a copy of the relevant guideline [15] and support in quality assurance for data collection (including reminder stickers and education). EDs randomised to the intervention group received the NET intervention—a complex, multi-faceted intervention that operated at the level of the clinician and the organisation. The NET intervention included the following components:

(i) Access to the guideline [21],
(ii) One stakeholder meeting per intervention group ED (1 h duration) between NET clinicians/researchers and local stakeholders (clinicians and management) to secure ‘buy-in’ at the ED level;
(iii) Identification of local opinion leaders (nursing and medical) via the key-informant method (with ED Directors or Senior Consultants being the key informant for each ED);
(iv) Delivery of an interactive train-the-trainer workshop to local opinion leaders (1 day duration) led by content experts and NET clinicians and incorporating information provision and skills training;
(v) Delivery of local workshops (20 min duration) by local opinion leaders to ED staff using provided materials; and
(vi) Provision of screening tools and information booklets in English and translated into five languages commonly spoken in Australia.

Intervention components were selected to target modifiable barriers and enablers for the implementation of key recommendations regarding the management of mTBI [13, 14]. The design of the NET intervention and its components have been described in detail elsewhere [2, 10, 14, 22]. The delivery of the NET intervention and resources required for each intervention component are described in detail in Additional file 1. Delivery of the comparator or control condition is described in detail in Additional file 2.

### Incremental effectiveness

The primary outcome for the main effectiveness analysis, appropriate PTA screening of patients with mTBI (based on chart review), was also specified as the primary outcome for the economic evaluation. Supplementary analyses were also conducted for a number of secondary outcomes. Provision of written information on discharge from the ED (INFO) and ‘safe discharge’ (SAFED, defined as CT scan appropriately provided plus PTA plus INFO) were specified as secondary clinical practice outcomes for economic evaluation in the NET sample. Patient-reported measures of anxiety (Hospital Anxiety and Depression Scale [23, 24]), post-concussive symptoms (13-item Rivermead [25]), and health-related quality of life (SF-12-based SF6D index scores [26–28])^1^ were specified as secondary patient outcomes for economic evaluation in the NET-Plus sample. Intervention effects with respect to ‘PTA’, ‘INFO’, ‘SAFED’, clinical patient outcomes, and health-related quality of life were estimated using generalised estimating equation (GEE) models as specified for the main effectiveness analysis (see [2], (Bosch M, McKenzie J, Ponsford JL, Turner S, Chau M, Tavender EJ, et al.: Evaluation of a targeted, theory-informed implementation intervention designed to increase uptake of emergency management recommendations regarding adult patients with mild traumatic brain injury: results of the NET cluster randomised controlled trial, Forthcoming)) in STATA/MP 13.1 for Windows [STATA Corp, 2016]. GEE models appropriately account for correlation of observations clustered within EDs.

### Incremental cost

For the NET sample, total cost per patient was calculated as the sum of per patient intervention costs (calculated at the ED level and averaged across patients in each ED) and the per-patient cost of medical and surgical services received in the ED/inpatient ward following their initial mTBI presentation (calculated at the patient level).^2^ For the NET-Plus sample, the total cost per patient additionally includes the per-patient cost of mTBI-related health service utilisation for a 4-week period, post-discharge from their initial mTBI presentation (calculated at the patient level).

Data sources for each component of total cost are summarised in Table 1 and described in detail in Additional files 1, 2, 3, 4, 5 and 6. Additional files 1 and 2 provide a detailed description of line items and data sources for delivery of the intervention and control conditions. Additional file 3 reproduces health service utilisation items from the chart audit used to estimate resource-use in the ED/inpatient ward. Additional file 4 reproduces health service utilisation items included in phone interviews with NET-Plus participants and used to estimate mTBI-related service utilisation in the post-discharge period. Tables A5-1 and A5-2 in Additional file 5 list unit costs used to convert physical units of resource use to dollar values for delivery of the intervention/control conditions and for health service utilisation, respectively.

Given the characteristic distribution of health costs (truncated at zero and right-skewed), the importance of obtaining readily interpretable marginal effects, and our interest in population-average effects, we modelled intervention effects on total costs using one-part generalised estimating equation (GEE) models with a log link rather than transformed ordinary least squares or two-part models [29]. Specification of the log link for the GEE models permits natural interpretation of marginal effects on cost without retransformation [29]. Correlation structure, standard errors, and controls for confounding variables were as specified for the main effectiveness analysis (see [2], (Bosch M, McKenzie J, Ponsford JL, Turner S, Chau M, Tavender EJ, et al.: Evaluation of a targeted, theory-informed implementation intervention designed to increase uptake of emergency management recommendations regarding adult patients with mild traumatic brain injury: results of the NET cluster randomised controlled trial, Forthcoming)). Aside from preliminary analyses of costs associated with the delivery of TTT workshops (conducted using Excel 2013), all analyses of total and incremental cost were conducted using STATA/MP 13.1 for Windows [STATA Corp, 2016].

### Adjustment for differential timing

All costs were expressed in 2015 AUD by attaching 2015 unit costs where available or inflating actual costs to December quarter 2015 using the all-items Consumer Price Index [30]. For the within-trial analysis presented here, all costs and consequences occurred within a 12-month period, and so, treatment effects were calculated for *undiscounted* costs and *undiscounted* benefits.

### Incremental cost-effectiveness

Results from the main analysis on the primary outcome were expressed as additional costs (savings) per patient appropriately screened for PTA. For secondary clinical practice outcomes, results were expressed as additional costs (savings) per patient who received patient information upon discharge and per patient safely discharged. For patient-reported health outcomes in the NET-Plus sample, results were expressed as additional costs (savings) per point difference on anxiety questions of Hospital Anxiety and Depression Scale, additional costs (savings) per point difference on the Rivermead Post-Concussive Symptoms (PCS) checklist, and additional costs (savings) per point difference in SF6D utility index scores. Point estimates for incremental cost-effectiveness were calculated as the average treatment effect on total cost per patient divided by the average treatment effect on the relevant outcome.

### Uncertainty

Parametric confidence intervals around the incremental cost-effectiveness ratio were derived via the application of Fieller’s Theorem using iprogs.do [31] in STATA/MP 13.1 for Windows [STATA Corp, 2016]. Non-parametric confidence intervals were derived by applying the acceptability method to a distribution of incremental cost-effectiveness ratios generated from bootstrap re-estimation of estimated treatment effects for incremental cost and incremental effectiveness (i.e. bootstrap re-estimation of results from main effectiveness models reported in Tables 3 and 4, with adjustment for minimisation factors and pre-specified confounders as appropriate). Non-parametric confidence intervals and bootstrap re-estimation were implemented using bsceaprogs.do and bmultiv.do [31] in STATA/MP 13.1 or 15.0 for Windows [STATA Corp, 2016; 2017].

Cost-effectiveness acceptability curves (CEACs) were used to visualise the uncertainty associated with the decision to replace the comparator with the evaluated intervention. CEACs plot the proportion of draws in the bootstrapped distribution of cost-effectiveness for which the intervention is cost-effective, compared to its comparator, at varying funding thresholds (also known as cost-effectiveness thresholds or reference ICERs). In other words, the CEAC tracks changes in the probability that the evaluated intervention is cost-effective as the funding threshold is increased/decreased. For the present study, CEACs were derived using bsceaprogs.do [31] based on data generated from bootstrap re-estimation of our estimates of incremental cost and incremental effectiveness using bmultiv.do [31] in STATA/MP 13.1 or 15.0 for Windows [STATA Corp, 2016; 2017].

Sensitivity analyses were completed using upper/lower bound estimates of uncertain parameters and varying potentially contentious assumptions. For example, the inclusion of urgency-related group (URG) costs for service use in the ED *and* costs of mTBI-related treatment in the ED for the base-case analysis carries a risk of double-counting; URG costs were therefore excluded in sensitivity analyses to test robustness to this decision. Along similar lines, the inclusion of diagnosis-related group (DRG) costs for service use during inpatient admissions *and* costs of mTBI-related treatment during inpatient admissions carries a risk of double-counting. However, the inclusion of DRG costs also carries the risk of an additional possible bias, arising from difficulties in identifying mTBI-related admissions and the potential for misclassification of a small number of high-cost admissions to exert undue influence on results. For this reason, DRG costs were excluded from the base-case analysis but included alongside costs of mTBI-related treatment during inpatient admissions in sensitivity analysis.

## Results

### Participants

A total of 1943 patients from 17 control and 14 intervention EDs (control: *n* = 1050; intervention: *n* = 893) were included in the analysis of clinical practice outcomes (NET sample). A total of 343 patients from 14 control and 10 intervention EDs (control: *n* = 218; intervention: *n* = 126) participated in follow-up interviews and were included in the analysis of patient-reported health outcomes (NET-Plus sample). Table 2 summarises ED and patient characteristics at baseline for treatment and control groups in both NET and NET-Plus samples.

**Table 2:** ED and patient characteristics

	*N* (%)/mean (SD)	*N* (%)/mean (SD)	*N* (%)/mean (SD)	*N* (%)/mean (SD)
	NET sample		NET-Plus sample	
	Control ^1^	Intervention ^2^	Control ^3^	Intervention ^4^
ED structural characteristics				
Hospital type (private)	1 (6%)	1 (7%)	1 (7%)	1 (10%)
Hospital type (public)	16 (94%)	13 (93%)	13 (93%)	9 (90%)
Trauma unit	3 (18%)	4 (29%)	2 (14%)	3 (30%)
Short stay unit	13 (76%)	10 (71%)	10 (71%)	6 (60%)
Annual presentation rate 2012, mean (SD)	44,710 (22593)	41,255 (16512)	44,592 (25046)	36,852 (16913)
Annual presentation rate 2012, median (IQR)	42,495 (34,313 to 46,690)	41,574 (27,075 to 55,667)	38,816 (32,833 to 52,963)	32,612 (25,646 to 47,189)
Existence of protocol for mTBI	4 (24%)	3 (21%)	3 (21%)	3 (30%)
NET-Plus	14 (82%)	13 (93%)	14 (100%)	10 (100%)
Rurality (regional)	7 (41%)	5 (36%)	6 (43%)	4 (40%)
Patient characteristics				
Age	50.9 (23.65)	54.2 (24.93)	53.5 (20.59)	55.2 (21.17)
Sex (% male)	476 (45%)	390 (44%)	105 (48%)	51 (41%)
After hours presentation	748 (71%)	653 (73%)	149 (68%)	90 (72%)
Initial GCS 15	961 (92%)	768 (86%)	213 (98%)	115 (92%)
Initial GCS 14	89 (8%)	125 (14%)	5 (2%)	10 (8%)
Mechanism of injury, incidental fall	492 (47%)	481 (54%)	113 (52%)	65 (52%)
Mechanism of injury, road traffic	58 (6%)	51 (6%)	11 (5%)	10 (8%)
Mechanism of injury, violence/assault	250 (24%)	163 (18%)	36 (17%)	15 (12%)
Mechanism of injury, sport	62 (6%)	55 (6%)	17 (8%)	11 (9%)
Mechanism of injury, others	179 (17%)	137 (15%)	41 (19%)	24 (19%)
Mechanism of injury, unclear/not reported	9 (0.9%)	6 (0.7%)	0 (0.0%)	0 (0.0%)
Presence other injuries (outside head)	508 (48%)	516 (58%)	105 (48%)	63 (50%)
Alcohol/illicit drug involvement	237 (23%)	206 (23%)	28 (13%)	15 (12%)
Pre-existing coagulopathy or anti-coagulant or anti-platelet drugs	175 (17%)	165 (18%)	37 (17%)	17 (14%)
Known previous neurological condition	202 (19%)	191 (21%)	26 (12%)	13 (10%)
Known neurosurgery	14 (1.3%)	18 (2.0%)	2 (0.9%)	3 (2.4%)
Scalp laceration	532 (51%)	464 (52%)	130 (60%)	67 (54%)
Scalp haematoma	400 (38%)	372 (42%)	79 (36%)	42 (34%)
Clinical suspicion of skull fracture	51 (4.9%)	57 (6%)	8 (3.7%)	8 (6%)
Loss of consciousness	186 (18%)	155 (17%)	50 (23%)	18 (14%)
Vomiting	56 (5%)	49 (5%)	12 (6%)	4 (3.2%)
Headache	259 (25%)	231 (26%)	44 (20%)	37 (30%)
Post traumatic seizure	3 (0.3%)	6 (0.7%)	0 (0.0%)	2 (1.6%)
Focal neurological deficit	21 (2.0%)	13 (1.5%)	5 (2.3%)	3 (2.4%)

^1^Number of patients = 1050; numbers of clusters = 17

^2^Number of patients = 893; number of clusters = 14

^3^Number of patients = 218; number of clusters = 14

^4^Number of patients = 125; number of clusters = 10

### Incremental effectiveness

Results regarding the effect of the NET intervention on clinical practice outcomes (based on chart audit for treated patients), proxy measures of clinical practice (based on clinician self-report and behavioural simulation), and clinical outcomes (based on patient self-report) have been reported in detail elsewhere (Bosch M, McKenzie J, Ponsford JL, Turner S, Chau M, Tavender EJ, et al.: Evaluation of a targeted, theory-informed implementation intervention designed to increase uptake of emergency management recommendations regarding adult patients with mild traumatic brain injury: results of the NET cluster randomised controlled trial, Forthcoming). Table 3 provides a brief summary of results from the main effectiveness analysis that underpin the economic evaluations reported here. On the primary outcome for the economic evaluation, appropriate PTA screening (based on chart review), patients treated in intervention EDs were 14% more likely to have been treated in line with guideline recommendations than were patients in control EDs (adjusted absolute risk difference 13.63%; 95%CI 8.3%, 19.0%; *p* < 0.001). On the secondary clinical practice outcome, provision of written patient information (INFO), intervention and control group patients faced about the same chance of being treated in line with guideline recommendations (adjusted absolute risk difference 3.15%; 95%CI − 3.0%, 9.3%; *p* = 0.316). For the composite indicator of clinical practice, ‘safe discharge’ (SAFED, defined as CT scan appropriately provided plus PTA plus INFO), patients treated in intervention group EDs were more likely to meet the criteria for ‘safe discharge’ (absolute risk difference 3.49%; 95% CI 1.0%, 6.0%; *p* = 0.0091); with this result being largely driven by the difference in appropriate PTA.

**Table 3:** Effect of the intervention on clinical practice and patient outcomes

Variable	No. of patients (EDs)		*N* (%)/mean (SE)		Increment, raw (95%CI)^	Increment, adjusted (95%CI)
	Rx	Control	Rx	Control		
Clinical practice outcomes (NET sample)						
PTA^1^	893 (14)	1050 (17)	117 (13.1%)	12 (1.1%)	11.96% (9.8, 14.1)	13.63% (8.3, 19.0)^†^
INFO	785 (14)	944 (17)	160 (20.4%)	175 (18.5%)	1.84% (− 1.9, 5.6)	3.15% (− 3.0, 9.3)^†^
SAFED^2^	402 (14)	413 (17)	14 (3.5%)	0 (0%)	3.49% (1.0, 6.0)	–
Clinical outcomes and quality of life (NET-Plus sample)						
Anxiety^3^	125 (10)	218 (14)	3.43 (0.32)	4.27 (0.27)	− 0.83 (− 1.69, 0.02)	− 0.52 (− 1.34, 0.30)^††^
Rivermead^4^	125 (10)	218 (14)	4.73 (0.49)	6.68 (0.59)	− 1.96 (3.65, 0.26)	− 1.15 (− 2.77, 0.48)^††^
HRQoL^5^	123 (10)	208 (14)	0.805 (0.01)	0.776 (0.01)	0.029 (− 0.00, 0.06)	0.030 (− 0.00, 0.06)^‡^

^^^Increment, raw = unconditional difference in absolute risk or average scores due to exposure to the intervention from two sample *t* test with equal variances

^†^Increment, adjusted = difference in absolute risk due to exposure to the intervention; adjusted for the following minimisation factors and pre-specified confounders: age, sex, out_of_hours, rurality, mTBI protocol, ED participation in NET-Plus, and annual_presentation_rate. Estimates derived from *margins, dydx(i.study_group)* after *xtgee, family(binomial) link(logit) corr(exchangeable) vce(robust)* to account for within-cluster correlation structure and yielding cluster-robust standard errors even if the correlation structure is misspecified

^††^Increment, adjusted = difference in average scores due to exposure to the intervention; adjusted for the following minimisation factors and pre-specified confounders: age, sex, out_of_hours, rurality, mTBI protocol, and annual_presentation_rate. Estimates derived from *margins, dydx(i.study_group)* after *xtgee, family(Gaussian) link(identity) corr(independent) vce(robust)* to account for within-cluster correlation structure and yielding cluster-robust standard errors even if the correlation structure is misspecified

‡Increment, adjusted = difference in average scores due to exposure to the intervention; adjusted for the following minimisation factors and pre-specified confounders: age, sex, out_of_hours, rurality, mTBI protocol, and annual_presentation_rate. Estimates derived from *margins, dydx(i.study_group)* after *xtgee, family(Gaussian) link(log) corr(independent) vce(robust)* to account for within-cluster correlation structure and yielding cluster-robust standard errors even if the correlation structure is misspecified

^1^Primary outcome for the economic evaluation

^2^Defined as PTA, INFO, and CT where CT denotes whether a CT scan was provided in the presence of a risk factor that justifies the scan (age 65 or older; GCS < 15; amnesia; suspected skull fracture; vomiting and coagulopathy) [26] (assessed in the cohort of patients for whom risk criteria were recorded only). CT therefore indicates whether a scan was appropriately provided but not whether a scan was ‘appropriately denied’. CT and SAFED only assessed in the cohort of patients for whom risk criteria were recorded

^3^Anxiety measured using the relevant questions in the Hospital Anxiety and Depression Scale giving a score between 0 and 21, higher scores indicate higher levels of anxiety

^4^Post-concussion symptoms measured using the 13-item Rivermead scale giving a score between 0 and 52, higher scores indicate greater severity of post-concussion symptoms

^5^SF-12v2-based SF6D index scores calculated using weights from Brazier and Roberts [20]. SF-12v2-based SF6D index scores range between 0.350 (the ‘pits’) and 1.000 (full health)

For clinical patient outcomes (NET-Plus sample), there was no statistically significant difference between intervention and control groups for anxiety (adjusted mean difference − 0.52; 95% CI − 1.34–0.30; *p* = 0.216) or post-concussion symptoms (adjusted mean difference − 1.15; 95% CI − 2.77–0.48; *p* = 0.167). Finally, SF6D HRQoL scores were higher in the intervention group than in the control group (adjusted mean difference 0.03; 95% CI − 0.00–0.06, *p* = 0.053). However, this difference fell just short of the pre-specified 0.05 level of statistical significance and it is not clear that the effect is sufficiently large to be considered clinically significant [32, 33].

### Incremental cost

The total cost of delivering the NET intervention to the 14 EDs and 893 patients included in the intervention group was just over $124,000 or $139 per patient. Total costs for each component of the NET intervention are detailed in Additional file 1: Table A1-1. The total cost of delivering the control condition to the 17 EDs and 1050 patients in the control group was just under $850 or less than $1 per patient. The total costs for each component of the control condition are detailed in Additional file 2: Table A2-1. These figures demonstrate the much larger upfront cost of the NET intervention (mean difference $138.20; 95% CI $135, $141; *p* < 0.000).

The total cost of medical and surgical services received in the ED/inpatient ward following the initial mTBI presentation was estimated at $826 in the intervention group and $777 in the control group (adjusted mean difference $23.86; 95% CI − $106, $153; *p* = 0.719). When combined with intervention delivery costs, the total cost of the NET intervention in the 2 months post-intervention was $965 as compared to $778 for the control condition (adjusted mean difference $169.89; 95% CI $43, $297; *p* = 0.009).

Results are qualitatively similar over the longer follow-up in the NET-Plus sample. The NET intervention does not appear to deliver savings in health service utilisation (adjusted mean difference $341.78 per patient; 95%CI − $58, $742; *p* = 0.094). Savings from lower health service utilisation are therefore unlikely to offset the significantly higher upfront cost of the intervention. Once again, estimates of the net effect of the intervention on total cost (intervention delivery cost net of health service utilisation) suggest that the intervention entails significantly higher costs than the control condition (adjusted mean difference $505.06; 95%CI $96, $915; *p* = 0.016). Table 4 summarises estimation of group totals and increments for intervention delivery, health service utilisation, and total cost.

**Table 4:** Effect of the intervention on Rx cost, HSU cost, and total cost (per patient)

Variable	No. of patients (EDs)		Mean (SE)		Incremental cost, raw (95%CI)^†^	Incremental cost, adjusted (95%CI)^‡^
	Rx	Control	Rx	Control		
NET						
Rx Cost						
Base-case	893 (14)	1050 (17)	$139.0 (1.6)	$0.8 (0.0)	$138.20 (135,141)	–
No URG	893 (14)	1050 (17)	$139.0 (1.6)	$0.8 (0.0)	$138.20 (135,141)	–
HSU Cost						
Base-case	893 (14)	1050 (17)	$825.9 (44)	$777.4 (33)	$48.52 (− 57, 154)	$23.86 (− 106,153)
No URG	893 (14)	1050 (17)	$354.0 (42)	$315.7 (31)	$38.35 (− 62, 139)	-$1.08 (− 125,122)
Total Cost						
Base-case	893 (14)	1050 (17)	$964.9 (44)	$778.2 (33)	$186.72 (81, 292)	$169.89 (43, 297)
No URG	893 (14)	1050 (17)	$493.0 (42)	$316.5 (31)	$176.55 (76, 277)	$159.96 (39, 281)
NET-Plus, including post-NET HSU						
Rx Cost						
Base-case	126 (10)	218 (14)	$143.8 (4.6)	$0.8 (0.0)	$142.96 (136,150)	–
No URG	126 (10)	218 (14)	$143.8 (4.6)	$0.8 (0.0)	$142.96 (136,150)	–
HSU Cost						
Base-case	126 (10)	218 (14)	$980.8 (235)	$738.5 (72)	$242.38 (− 156,640)	$341.78 (− 58, 742)
No URG	126 (10)	218 (14)	$526.6 (233)	$276.5 (66)	$250.06 (− 137,638)	$338.54 (− 56, 733)
Total Cost						
Base-case	126 (10)	218 (14)	$1124.6 (235)	$739.3 (72)	$385.34 (− 12, 783)	$505.06 (96, 915)
No URG	126 (10)	218 (14)	$670.3 (233)	$277.3 (66)	$393.02 (− 6, 780)	$543.49 (116, 971)

†Incremental cost, raw = unconditional difference in cost per patient due to exposure to the intervention from two sample *t* test with equal variances

‡Incremental cost, adjusted = difference in cost due to exposure to the intervention; adjusted for the following minimisation factors and pre-specified confounders: age, sex, out_of_hours, rurality, mTBI protocol, ED participation in NET-Plus, and annual_presentation_rate. Estimates derived from *margins, dydx(i.study_group)* after *xtgee, family(gamma) link(log) corr(exchangeable) vce(robust)* to account for within-cluster correlation structure and yielding cluster-robust standard errors even if the correlation structure is misspecified

### Incremental cost-effectiveness

The base-case analysis on the primary outcome suggests that the NET intervention is more costly and more effective than passive dissemination, entailing an additional cost of $1246 per additional patient appropriately screened for PTA ($169.89/0.1363). Confidence intervals around the base-case point estimate suggest that—given a sufficiently high funding threshold for the outcome of interest—we can be at least 95% confident that the NET intervention represents good value in comparison to passive dissemination. Upper confidence limits from parametric and non-parametric 95%CIs are $2055 and $2066 respectively, and so, if the funding threshold sits at around $2000 per patient appropriately screened for PTA, then we can be confident that NET delivers good value.

At lower funding thresholds, this finding is reversed and we can be 95% confident that the NET is not cost-effective when compared against passive dissemination. Lower confidence limits from parametric and non-parametric 95%CIs are $525 and $595 respectively, and so, if the funding threshold sits at around $500 per patient appropriately screened for PTA, then we can be 95% confident that passive dissemination delivers better value than the NET intervention. Table 5 summarises results from the cost-effectiveness analysis, including parametric and non-parametric 95%CIs.

**Table 5:** Incremental cost-effectiveness for clinical practice and patient outcomes

Variable	ΔC	ΔE	ΔC / ΔE	Parametric (95%CI)^†^	Non-parametric (95%CI)^‡^
Clinical practice outcomes (NET sample)					
PTA^1^	$169.89 (43, 297)	0.1363 (0.083, 0.190)	$1246	($525, $2055)	($595, $2066)
INFO^2^	$169.89 (43, 297)	0.0315 (− 0.030, 0.093)	$5393	($1672,− $18,125)	($1909, − $22,865)
SAFED^3^	$169.89 (43, 297)	0.0349 (0.010, 0.060)	$4868	($1882, $11928)	($2093, $11574)
Clinical outcomes and quality of life (NET-Plus sample)					
HADS Anxiety^4^	$505.06 (96, 915)	− 0.52 (− 1.34, 0.30)	$971^	($132, − $1086)	($215, − $1084)
Rivermead PCS^5^	$505.06 (96, 915)	− 1.15 (− 2.77, 0.48)	$441^	($54, − $1270)	($90, − $1346)
SF6D HRQoL^6^	$505.06 (96, 915)	0.030 (− 0.00, 0.06)	$16,948	($2015,-$367,759)	($3796,-$314,637)

^†^Parametric CIs derived via Fieller’s Theorem using iprogs.do [25] based on treatment effects reported in Tables 3 and 4 and using standard errors and correlations calculated from non-parametric bootstrap re-estimation

^‡^Non-parametric CIs derived via acceptability method using bsceaprogs.do [25] based on the data generated from bootstrap re-estimation of our estimates of incremental cost and incremental effectiveness using bmultiv.do [25]

^1^Cost per patient appropriately screened for PTA (primary outcome)

^2^Cost per patient who received written patient information upon discharge (INFO)

^3^Cost per safe discharge (SAFED) where SAFED defined as CT scan appropriately provided plus PTA plus INFO

^4^Cost per point improvement on anxiety questions of Hospital Anxiety and Depression Scale (HADS), higher scores indicate higher levels of anxiety

^5^Cost per point improvement on the Rivermead post-concussive symptoms scale, higher scores indicate greater severity

^6^Cost per point improvement in SF6D index scores, higher scores indicate higher HRQoL

^^^Calculated as ΔC/− ΔE to reflect the fact that higher scores indicate greater severity for HADS and Rivermead

**Fig. 1:**
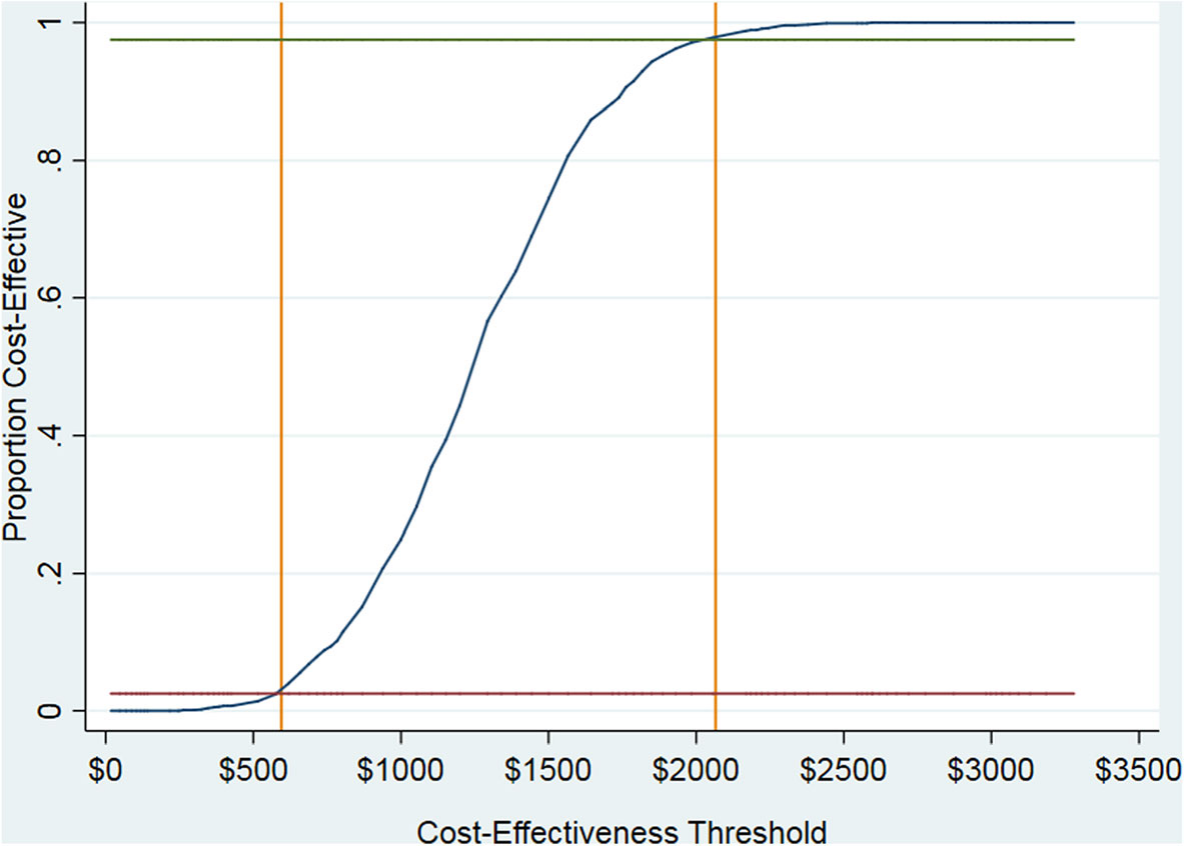
Cost-effectiveness acceptability curve for the primary outcome (PTA)

Figure 1 gives the cost-effectiveness acceptability curve (CEAC) for the primary outcome. The CEAC (solid blue line) plots the relative frequency, or probability, that the NET intervention is cost-effective, compared to passive dissemination at varying levels of the funding threshold expressed in terms of cost per additional patient appropriately screened for PTA. The green and red horizontal lines intersect the CEAC where the NET intervention is cost-effective for 97.5% and 2.5% of the distribution respectively, at funding thresholds of $2066 and $595 per patient appropriately screened for PTA (corresponding with upper and lower confidence limits estimated via the acceptability method). For intermediate thresholds (*between* the upper and lower confidence limits), we can no longer be so confident that one or other of the two alternatives represents better value for money. For decision-makers interested in lower levels of confidence, NET is the most cost-effective alternative for over 50% of the distribution if the funding threshold exceeds $1250 per patient appropriately screened for PTA.

**Fig. 2:**
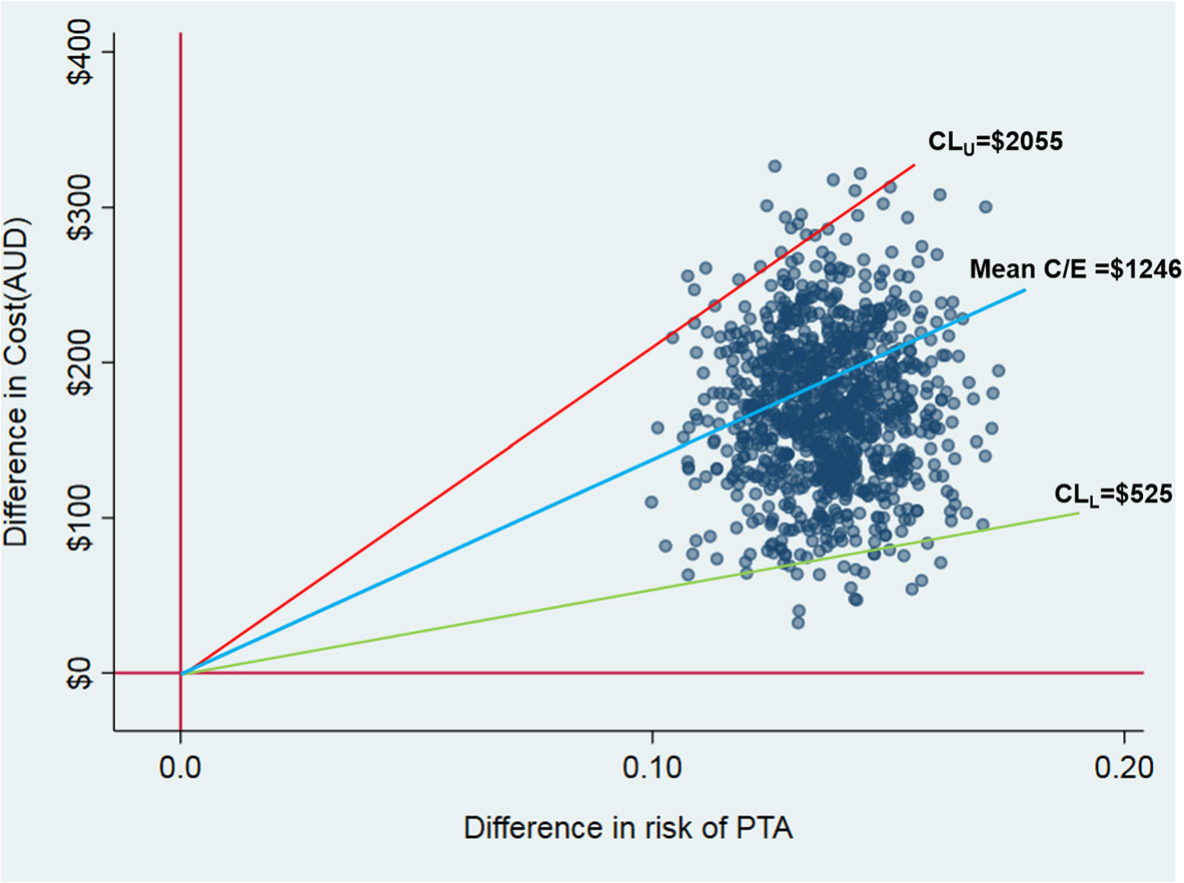
Cost-effectiveness plane and cost per additional patient appropriately screened for PTA (primary outcome)

**Fig. 3:**
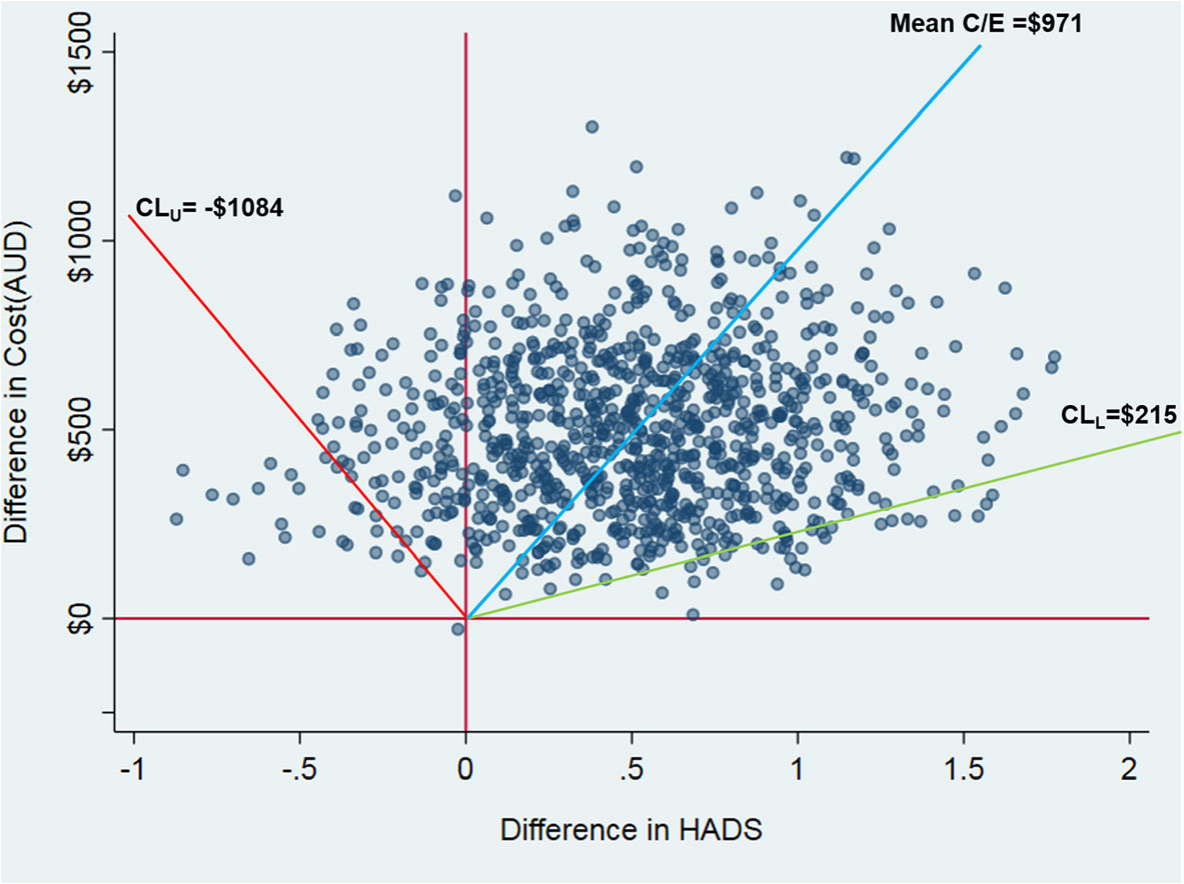
Cost-effectiveness plane and cost per point improvement on HADS

For PTA and SAFED, we have pattern 1 findings, with the scatter of cost-effect pairs on the cost-effectiveness plane restricted to the northeast (NE) quadrant indicating that the NET intervention is statistically significantly more costly and more effective than passive dissemination [31]. Figure 2 shows the scatter on the cost-effectiveness plane for PTA, providing a graphical description of pattern 1 findings. For INFO, HADS Anxiety, Rivermead PCS, and SF6D HRQoL, we have pattern 2 findings, with the scatter of cost-effect pairs on the cost-effectiveness plane spanning the NE and northwest (NW) quadrants indicating that the NET intervention carries a statistically significantly greater cost than passive dissemination but with no significant difference on the relevant outcome [31]. For pattern 2 findings, the point estimate appears to sit outside the upper and lower confidence limits reported in Table 5, an artefact arising from discontinuities in cost-effectiveness ratios as we move between quadrants on the cost-effectiveness plane. Figure 3 demonstrates the relative position of the point estimate, the scatter, and 95% confidence limits on the cost-effectiveness plane for cost per point improvement on HADS Anxiety and confirms that the point estimate is bounded by upper and lower confidence limits for pattern 2 findings.

For INFO, HADS Anxiety, Rivermead PCS, and SF6D HRQoL, there is no funding threshold at which we can be 95% confident that the NET intervention represents good value compared with passive dissemination. However, given a sufficiently low funding threshold for the outcome of interest, we can be at least 95% confident that the NET intervention represents poor value in comparison to passive dissemination. For HADS, lower confidence limits from parametric and non-parametric 95%CIs are $132 and $215 respectively, and so, if the funding threshold sits at around $100 per point improvement on the HADS, then we can be 95% confident that passive dissemination delivers better value than the NET intervention.

For decision-makers interested in lower levels of confidence, it is still possible to identify a funding threshold for INFO, HADS Anxiety, Rivermead PCS, and SF6D HRQoL at which NET is the most cost-effective alternative for a proportion of the distribution (e.g. for over 50% and 80% of the distribution). NET is the most cost-effective alternative for over 50% of the distribution if the funding threshold exceeds $17,000 per point improvement in SF6D index scores, and for over 80% of the distribution if the funding threshold exceeds $34,000 per point improvement in SF6D index scores. For other outcomes, NET was the most cost-effective alternative with 80% probability if the funding threshold exceeds $13,000 per additional patient provided with written patient information, $4500 per point improvement on HADS, and $1000 per point improvement on the Rivermead. Full results available upon request.

### Sensitivity analysis

Table 5 reports results from the cost analysis after excluding URG costs to test sensitivity to double counting where, in the base-case, URG costs for service use in the ED (less average imaging costs) have been included alongside costs of mTBI-related treatment in the ED. Results after excluding URG costs are qualitatively identical to results from the base-case analysis. On the primary outcome, the NET intervention is more costly and more effective than the passive dissemination of the relevant guideline (ΔC/ΔE = $159.96/0.1363 = $1174). Confidence intervals around this point estimate suggest that—provided that the funding threshold is above $1958 per additional patient appropriately screened for PTA—we can be at least 95% confident that the delivery of the NET intervention represents good value in comparison to passive dissemination. If, on the other hand, the funding threshold for the primary outcome is less than $469 per additional patient appropriately screened for PTA, then we can be 95% confident that the NET intervention is less cost-effective than passive dissemination. NET is the most cost-effective alternative for over 50% of the distribution if the funding threshold exceeds $1200 per additional patient appropriately screened for PTA.

While results were insensitive to inclusion/exclusion of URG costs, the same cannot be said for inclusion/exclusion of diagnosis-related group (DRG) costs to capture the cost of inpatient admissions. In the base-case analysis, the total cost of the NET intervention in the 2 months post-intervention was $965 as compared to $778 for the control condition (adjusted mean difference $169.89; 95% CI $43, $297; *p* = 0.009). Including DRG costs alongside costs of mTBI-related treatment during inpatient admissions had the effect of increasing group averages ($1838 versus $1385) and inflating incremental cost (adjusted mean difference $452.91; 95% CI $150, $756; *p* = 0.003), with predictable consequences for incremental cost-effectiveness across all primary and secondary outcomes. Full results from the sensitivity analyses are available on request.

## Discussion

Results suggest that the NET intervention is more costly and more effective than passive dissemination for improving management of mTBI in the ED and that—*given sufficiently high funding thresholds for improvements in PTA and safe-discharge*—we can be at least 95% confident that the NET intervention represents good value in comparison to passive dissemination. Direct estimates of funding thresholds for PTA and safe discharge are not available and it was not feasible to derive our own estimates as part of the NET study. Further thought is therefore required in order to translate our findings into recommendations for policy and practice.^3^

Funding thresholds for more commonly used measures of health gain such as quality-adjusted life years (QALYs) have previously been estimated or proposed for different jurisdictions but vary widely and the level and meaning of these funding thresholds has recently become contested [34, 35].^4^ For any funding threshold expressed in terms of cost per QALY gained, we can calculate ‘bounds’ on the relationship between PTA and QALYs that would need to hold in order for NET to be considered cost-effective. Moreover, we can conduct this exercise for different levels of the cost per QALY threshold in order to evaluate the sensitivity of our findings to an uncertain threshold. In order for the NET intervention to be considered cost-effective with 95% confidence at a funding threshold of $50,000 per QALY, each additional patient appropriately screened for PTA would need to generate ≥ 0.04 additional QALYs (at least 2 weeks of additional life expectancy in full health).^5^ For a higher (but still plausible) threshold of $100,000 per QALY, each additional patient appropriately screened for PTA would need to generate ≥ 0.02 additional QALYs (at least 1 week of additional life expectancy in full health). For a lower threshold (closer to recent estimates) of $25,000 per QALY, each additional patient appropriately screened for PTA would need to generate ≥ 0.08 additional QALYs (nearly 4.5 weeks of additional life expectancy in full health). Similar trade-offs can be calculated for other outcomes and at other confidence levels.

To put this in context, recent estimates of the population burden of disease for New Zealand suggest that mTBI imposes a total burden of 3277 years of life lost due to disability [36]. Assuming mTBI accounted for 70% of the 527,400 New Zealanders who had experienced at least one TBI, mTBI imposes an average burden of less than 3 days of life lost due to disability per mTBI patient [36]. The average per patient burden of mTBI is therefore *less than* the average per patient health gain that would have to be generated in order for us to be 95% confident that the NET intervention is cost-effective.

For SF6D index scores, we can obtain a more direct comparison against funding thresholds expressed in cost per QALY terms. While SF6D index scores were not explicitly combined with duration to calculate QALYs, we can calculate an implicit threshold per point improvement in SF6D index scores under different assumptions regarding the duration over which observed SF6D index scores persist and compare this to funding thresholds of $50,000 and $100,000 per QALY. In order for the NET intervention to be considered cost-effective with 80% confidence, the SF6D index scores observed in the NET-Plus sample would need to persist for more than 8 months if we assume a funding threshold of $50,000 per QALY, for more than 4 months if we assume a funding threshold of $100,000 per QALY, and for more than 16 months if we assume a funding threshold of $25,000 per QALY.^6^

In interpreting our results, several issues should be borne in mind. First, the NET study and main effectiveness analyses had both strengths and limitations that have been documented elsewhere (Bosch M, McKenzie J, Ponsford JL, Turner S, Chau M, Tavender EJ, et al.: Evaluation of a targeted, theory-informed implementation intervention designed to increase uptake of emergency management recommendations regarding adult patients with mild traumatic brain injury: results of the NET cluster randomised controlled trial, Forthcoming). To the extent that the study design and main effectiveness results underpin the cost-effectiveness analyses reported here, then our findings regarding cost-effectiveness are subject to the strengths and limitations of the NET study and main effectiveness analyses. Key strengths of the NET study with particular relevance to the cost-effectiveness analysis include the use of a systematic process to design a multi-faceted intervention, objective measures of clinical practice, and collection of data to inform a process evaluation. With regards to limitations of the NET study, the difficult task of identifying patients for inclusion in chart audit and then recruiting patients into the NET-Plus sample resulted in missing data on secondary health outcomes and raised the potential for selection bias. Along similar lines, secondary clinical outcomes could only be assessed for a subset of patients, potentially resulting in selection bias. While results from supplementary sensitivity analyses (see Additional file 7) suggest that findings from the economic evaluation reported here are robust to any such selection bias, results for secondary outcomes are for a subset of the full study sample and should be interpreted with caution.

Second, the present study estimates the relative cost-effectiveness of the intervention as implemented and may not reflect the relative cost-effectiveness under a wider roll-out. The NET intervention operates on a train-the-trainer model and relies on participation at the organisation level (to permit access to ED staff), at the ED level (to identify local opinion leaders), at the level of local opinion leaders (to attend TTT workshops and deliver local workshops), and at the clinician level (to attend local workshops). While participation in the context of the trial was not always consistent with maximising the effectiveness of the intervention,^7^ some level of participation was achieved at the organisation, ED, opinion-leader, and clinician levels in the majority of intervention group EDs. This may not be the case in the broader sample of Australian EDs meeting our inclusion/exclusion criteria, some or all of which may be targeted in a wider roll-out of the NET intervention.

While a lower level of participation in any wider roll-out might be expected to render the NET intervention less cost-effective, it is also possible that a NET intervention that has been scaled and streamlined ready for wider roll-out may be more cost-effective than the NET intervention as delivered in the trial. Of particular note, the TTT workshops accounted for over $70,000 or 56% of the total cost of delivering the NET intervention to the 14 intervention group EDs participating in the NET trial. While scale-up may require delivery of additional TTT workshops and would necessitate attendance by local opinion leaders from a larger number of EDs, there may also be scope to increase the number of local opinion leaders trained per workshop. That is to say, it may be possible to double the number of EDs attending the TTT workshop component of the NET intervention at much less than double the estimated cost of delivery.^8^ Moreover, there may be scope to reduce travel time and direct travel costs where scale-up requires delivery of additional TTT workshops. For example, TTT workshops could be scheduled at a wider range of locations or over a wider range of dates, allowing local opinion leaders to attend a TTT workshop in their home city rather than interstate. Finally, there may be scope to dramatically reduce the cost of TTT workshops using different modes of delivery such as live webinars and/or distribution of digital recordings, potentially resulting in a better balance between costs and effects.

Third, our estimate of incremental cost excludes differences in health service utilisation that may persist beyond the time horizon for the NET-Plus sample. Extending our analysis out to the longer follow-up in the NET-plus sample entailed a threefold increase in average incremental cost, and it is possible that we would see a further divergence between intervention and control groups over an even longer time horizon. Alternatively, the observed intervention effect with respect to health service utilisation may diminish over time; though it is unlikely that we would see a reversal in the observed trend (i.e. *lower* rather than higher health service utilisation in the treatment group) that would invalidate the results reported here. Nonetheless, the availability of multiple time-points for patient-reported health service utilisation and patient-reported health outcomes in the NET-Plus sample would have allowed us to provide a more complete exploration of the sensitivity of our findings to changes in time horizon.

Fourth, we observed treatment effects with respect to clinical practice and health outcomes only in the study sample and only during the study period. Cost-effectiveness may have been underestimated if treatment effects with respect to clinical practice and health outcomes were also incident upon patients who were treated in intervention-group EDs during the study period but who were not included in the study sample. Similarly, cost-effectiveness may have been underestimated if improvements in patient management observed during the study period persisted beyond the 2-month window from which eligible patients were selected for chart audit and subsequent follow-up of patient-report health outcomes. It may not be immediately apparent how such a bias could arise. The NET intervention operates at the level of the clinician and the organisation, and so intervention costs are semi-fixed in the sense that the cost of delivering treatment effects to one patient (as a consequence of providing all intervention components to one clinician in one ED) is exactly the same as the cost of delivering treatment effects to the total number of mTBI patients treated by one clinician in one ED (for as long as any behaviour change persists). Cost per patient is therefore a function of the number of patients in receipt of the treatment effect, and it may be possible to spread semi-fixed costs over a much larger number of patients than that included in the study sample. If, for example, improvements in patient management due to the intervention could be maintained for several years into the future (i.e. for the 14 intervention-group EDs), then many more patients would receive the benefits of these improvements without the need for any further investment. In this scenario, cost per patient would fall but treatment effects per patient would remain much the same, resulting in a better balance between costs and effects.

Finally, interpretation of results is complicated by the lack of widely accepted funding thresholds for the primary and secondary outcomes. While patient-reported outcomes in the NET-Plus sample included SF12-based SF6D index scores, we were unable to identify secondary data mapping the time path of HRQoL in mTBI patients and decided a priori of commencement of the study not to calculate QALYs by combining SF6D index scores with duration. For clinical practice outcomes and clinical health outcomes, a direct estimate of the funding threshold may have facilitated the interpretation of cost-effectiveness results. We were not able to identify direct estimates of the threshold in the literature, and it was not feasible to derive our own estimates as part of the NET study. In recognition of such difficulties, we make significant efforts to quantify and illustrate decision uncertainty and to couch results in terms of familiar funding decisions (such as whether or not to fund an intervention at a given cost per QALY). Such efforts allowed us to draw clear policy recommendations from our findings despite the lack of widely accepted funding or cost-effectiveness thresholds for the primary and secondary outcomes.

## Conclusion

The substantial upfront cost of the NET intervention was not offset by savings in health service utilisation and the NET intervention would therefore need to deliver significant improvements in clinical practice and/or patient outcomes in order for it to be considered cost-effective. As delivered in the trial, the NET intervention does appear to improve management of mTBI in the ED but funding thresholds for these improvements would need to be set implausibly high in order to justify the additional cost of the intervention. This finding serves as a timely reminder that initiatives to increase adherence to guideline recommendations should themselves offer good value for money.

The NET intervention was designed to maximise effectiveness subject to organisational constraints. Further gains in effectiveness may therefore be difficult to achieve without relaxing these organisational constraints. However, it may be possible to achieve a better balance between costs and outcomes by scaling and streamlining the NET intervention, potentially achieving the same or smaller changes in clinical practice but at a lower cost. This has broader implications for the design of implementation interventions—perhaps suggesting greater consideration should be given to smaller bang for (smaller) buck strategies.

## Additional files

Additional file 1: Appendix 1 Cost analysis for delivery of the NET intervention. (DOC 193 kb)

Additional file 2: Appendix 2 - Cost analysis for delivery of the comparator intervention. (DOC 98 kb)

Additional file 3: Appendix 3 - Chart audit health service utilisation items. (DOC 137 kb)

Additional file 4: Appendix 4 - Post-discharge health service utilisation items. (DOC 105 kb)

Additional file 5: Appendix 5 - Unit costs by category of resource use. (DOC 235 kb)

Additional file 6: Appendix 6 ISPOR Consolidated Health Economic Evaluation Reporting Standards (CHEERS) Checklist. (PDF 241 kb)

Additional file 7: Appendix 7 - Sensitivity analyses: potential impact of missing data and selection bias. (DOC 106 kb)

## Abbreviations

AUD: Australian dollar
CEAC: Cost-effectiveness acceptability curve
CPG: Clinical practice guideline
CRT: Cluster randomised trial
CT: Computed tomography
ED: Emergency department
GCS: Glasgow Coma Scale
HRQoL: Health-related quality of life
ICC: Intra-cluster correlation
ICERs: Incremental cost-effectiveness ratios
INFO: Provision of written patient information upon discharge
mTBI: Mild traumatic brain injury
NET: Neurotrauma Evidence Translation
PCS: Post-concussive symptoms
PTA: Post-traumatic amnesia
QALY: Quality-adjusted life year
SAFED: Safe discharge
TBI: Traumatic brain injury

## Footnotes

1 Preference-based HRQoL weights from the SF12-based SF6D must be combined with a period of time to permit estimation of intervention effects in QALY terms (and incremental cost-effectiveness in cost per QALY terms). For the NET study, SF12 data was available for a single time point, some 7 months post-discharge (on average). In the absence of repeated observation on HRQoL, we will treat measures of HRQoL as a summary measure of the effect of the intervention with respect to anxiety, sleep, functioning, and adverse effects at a single point in time and we make no attempt to combine SF6D index scores with time for the purposes of calculating QALYs.

2 The protocol specified a time horizon for the NET-Plus sample to coincide with the final follow-up for the NET-Plus sample, originally planned to take place at 3 months post-injury. Due to delays in recruitment, follow-up for NET-Plus patients occurred much later and over a much longer period than planned (mean = 210 days post discharge; SD 38.5 days; IQR 181–239; min = 130, max = 321). In such circumstances, using patient self-report on a 4-week window of health service utilisation to calculate per diems and then multiplying out over the entire period from discharge to follow-up would amplify any measurement errors due to recall bias or timing. We therefore treat patient self-report on health service utilisation as representative of a 4-week period, post-discharge from their initial mTBI presentation and take this period as the time horizon for economic evaluations in the NET-Plus sample.

3 Recommendations to fund a new intervention or change clinical practice are only consistent with maximising population health if they deliver a unit of the outcome of interest at a better price than is on offer elsewhere (i.e. better than interventions that are not or will not be funded if the new intervention or policy change is recommended). Decision-makers therefore need to know the price at which they can buy a unit of health outcome via the evaluated intervention or policy change (captured by the incremental cost effectiveness ratio) *and* the price at which alternative interventions could deliver that same health outcome (captured by the funding threshold, also known as the cost-effectiveness threshold or reference ICER). As Edney et al. [34] put it, “funding of a new technology with an ICER greater than the reference ICER results in a net decrease in population health, as represented by net QALY losses” (p248).

4 Using a new approach, Edney et al. [34] estimated the reference ICER for the Australian health care system at AUD28033 (95%CI AUD20758–AUD37667). This is broadly consistent with estimates for Australia (95%CI AUD28904, AUD51470 at current exchange rates) reported by Woods et al. [37] but much lower than the ballpark figure of AUD50000 per QALY that has been suggested as a rule-of-thumb for decision-makers [38] and much lower than previous estimates based on stated and revealed preference methods. For example, George et al. [39] estimated the threshold or reference ICER at between AUD42000 and AUD76000 using past funding decisions to intuit the revealed preferences of decision-makers. Shiroiwa et al. [40] estimated the funding threshold using a stated preference approach (maximum social willingness to pay per QALY) at between AUD64000 and AUD89000 (with higher/lower estimates for different sub-populations).

5 Calculated by dividing the funding threshold expressed in cost per QALY terms by the upper confidence limit on the cost-effectiveness ratio for the outcome of interest. For cost per additional patient appropriately screened for PTA, this calculation is $50,000/$2055 for the parametric 95%CI and $50,000/$2066 for the non-parametric 95%CI (see Table 5 and accompanying discussion). This calculation gives us 24.33 additional patients appropriately screened for PTA per QALY (or 0.04 QALYs per patient appropriately screened for PTA).

6 NET is the most cost-effective alternative for over 80% of the distribution if the funding threshold exceeds $34,000 per point improvement in SF6D index scores (where a point improvement is a full 0 (death) to 1 (full health) improvement at a single point in time). The ratio of cost per point improvement in SF6D index scores and cost per QALY ($34,000/$50,000) gives us the duration (fraction of a year) over which the SF6D index scores observed in the NET-Plus sample would have to persist in order for $34,000 per point improvement in SF6D index scores to be equivalent to a threshold of $50,000 per QALY. This calculation reflects the fact that QALYs are the product of duration (in years) and SF6D index scores, with one QALY being equivalent to 1 year with an SF6D score equal to one.

7 Only 60% of surveyed clinicians in intervention group EDs claimed to have attended or watched a PTA session, only 30% attended or watched CT session, and only 55% attended or watched session on handing out patient information.

8 Doubling the cost of attendance time, travel time and direct travel costs, and the cost of consumables for local opinion leaders (to accommodate participation from 28 rather than 14 EDs) but leaving other costs associated with delivery of the TTT workshops unchanged would see the total cost of delivering the TTT workshops increase to around $108,000 per 28 EDs, far less than double the cost of delivery to 14 EDs. This would equate to a decrease in the cost per ED from $5004 per ED (for 14 participating EDs) to $3870 per ED (for 28 participating EDs).
