# Supplementary material for: Economic evaluation of the NET intervention versus guideline dissemination for management of mild head injury in hospital emergency departments
Source: Implement Sci. 2018 Dec 5;13:147. doi: 10.1186/s13012-018-0834-6 (PMC6280545; doi:10.1186/s13012-018-0834-6)
Supplement: Supplementary file 1 — Appendix 1 Cost analysis for delivery of the NET intervention. (DOC 193 kb) [file 13012_2018_834_MOESM1_ESM.doc]

**Appendix 1 - Cost analysis for delivery of the NET intervention**

The NET intervention is a targeted, multi-faceted, theory-informed strategy to increase uptake of three key recommendations from the guideline. The NET intervention consisted of (i) access to the guideline, (ii) one stakeholder meeting between NET clinicians/researchers and local stakeholders (clinicians and management) per intervention group ED (one hour duration) to secure ‘buy-in’ at the organisational level; (iii) identification of local opinion leaders (nursing and medical) via the key-informant method (with ED Directors or Senior Consultants being the key informant for each ED), (iv) delivery of an interactive train-the-trainer workshop to local opinion leaders (one day duration) led by content experts and NET clinicians, and incorporating information provision and skills training; (v) delivery of local workshops (20 minute duration) by local opinion leaders to ED staff using provided materials; and (vi) provision of screening tools and information booklets in English and translated into five languages commonly spoken in Australia.

Fourteen EDs received the NET intervention. For intervention group EDs, 288 medical staff and 498 nursing staff participated in collection of clinician level data but this is a subset of clinicians exposed to the intervention. For locally delivered components of the NET intervention, resource use depends upon attendance and time commitment by NET and ED personnel. Resource use associated with the delivery of the NET intervention has been estimated from administrative and financial records. For example, project staff maintained detailed administrative records regarding attendance and time commitment by NET and ED personnel for components (ii), (iii), and (iv) above. For the purposes of evaluating fidelity to the intervention protocol for component (v) of the intervention, local opinion leaders maintained detailed records of preparation and delivery time for local workshops, content delivered at each workshop, and attendance at these workshops by ED staff. Table A1-1 summarises the resource-based costing for delivery of each component of the NET intervention. Further explanation regarding data sources and assumptions in estimating the cost of the NET interventions is provided below.

**Administration / coordination:** Resource-use associated with coordination of the NET intervention can be attributed to personnel and non-research overheads (e.g. office space).

- *NET Personnel:* The NET intervention ran over a period of eight months from commencement of the Stakeholder Meetings in late May 2014 to delivery of the last Local Workshop in January 2015. However, the Local Workshops were designed to run independently and the majority of activity associated with coordination of the NET intervention was concluded upon distribution of materials for Local Workshops in early August 2014 shortly after delivery of the Train-the-Trainer Workshops. Personnel costs associated with coordination of the NET intervention are therefore limited to a period of 10 weeks from late May 2014 to early August 2014. The NET intervention was coordinated by the NET Project Officer (NPO) and the NET Administrative Officer (NAO), with duties including liaison with venues, facilitators, and participants regarding availability and arrangements for workshops and meetings and production/distribution of relevant tools and materials. While both the NPO and NAO were employed full-time over the 10 week coordination period, a significant amount of their time was spent on tasks associated with intervention development and research / evaluation (both of which are excluded from the cost-analysis reported here). Moreover, the cost analysis attributes project officer and administrative officer time to each intervention component (e.g. Access to the Guideline, Stakeholder Meetings, Identifying Local Opinion Leaders, TTT Workshops, Local Workshops) where administrative/fidelity data identified hours spent on activities specific to the relevant intervention component (see below). For the NPO, administrative records attributed approximately 60% of an EFT over the 10 week coordination period to delivery of specific intervention components and intervention development. For the NAO, administrative records attributed approximately 40% of an EFT over the 10 week coordination period to delivery of specific intervention components and intervention development. We assume that any time not attributed to delivery of intervention components or intervention development can be attributed to either research / evaluation or to administration / coordination of the intervention (40% EFT for the NPO; 60% EFT for the NAO). In the base case, we attribute half of this remainder to research / evaluation and half to admin / coordination of the intervention (20% EFT for the NPO; 30% EFT for the NAO).
- *Overheads:* Costs associated with office space and utilities were estimated using the Monash University internal cost recovery formula for central services based on the maximum allowance in square metres for staff with the same level and function as the NPO and NAO (6m^2^ per Research Fellow plus 6m^2^ for supporting Administrative Staff). For simplicity, we cost the full 12m^2^ under the conservative assumption that 30% EFT is attributable to coordination and delivery of intervention components.

**Access to the guideline:**

Clinical leads working within EDs randomized to the *control group* were emailed an electronic link to the [*Initial Management of Closed Head Injury in Adults Guideline*](http://www.aci.health.nsw.gov.au/__data/assets/pdf_file/0003/195150/Closed_Head_Injury_CPG_2nd_Ed_Full_document.pdf) and instructed to ‘do whatever they would normally do when they became aware of a guideline’. In contrast, medical and nursing leads working within EDs randomized to the *intervention group* received the guideline on USB sticks (together with electronic copies of other tools and materials) during Train-the-Trainer workshops (for wider dissemination/implementation via the Local Workshops). Resource-use associated with access to the guideline in the intervention group has been captured in the cost analysis for TTT and Local Workshops and has not been separately costed in order to avoid double-counting. Costs associated with development and standard dissemination of the CPG outside of the NET trial are common and invariant to both treatment and control groups and, on this basis, have not been explicitly included in the cost analysis reported here.

**Stakeholder Meetings:** Of the fourteen EDs randomised to the intervention group, eight received the stakeholder meetings as planned. Meetings took place in from late May 2014 to mid-July 2014. Difficulties coordinating the diaries of key stakeholders precluded conduct of meetings for the remaining six intervention group EDs. We attach a zero cost for stakeholder meetings in EDs that did not receive a stakeholder meeting because any costs associated with failed attempts to schedule a meeting in these EDs are small in magnitude and reflected in the administration and coordination costs described above. Resource-use associated with delivery of stakeholder meetings at other EDs can be attributed to personnel, stakeholder time, venues, communications, and travel time.

- *NET Personnel:* Stakeholder meetings were generally facilitated by a NET investigator and the NET project officer. However, for two of the meetings, none of the NET investigators were available and the NET project officer facilitated these meetings with the assistance of the NET administration officer. Each workshop entailed discussion of: key recommendations from the guideline, supportive evidence, potential local barriers to implementation of key recommendations, and strategies for overcoming barriers to implementation. Most meetings ran for 60 mins duration (mean: 62.5 mins, range: 50 mins – 90 mins) but each meeting also entailed preparation time on the part of facilitators. Delivery and preparation time for the stakeholder meetings are costed at twice the sessional academic rate for a basic lecture (2 hours preparation time per 1 hour of delivery time) to reflect the use of two facilitators per meeting.
- *Stakeholder time:* Attendance by stakeholders varied across EDs, ranging from two to six ED staff (mean=3.75) and with a mix of medical and nursing staff in attendance. For example, one meeting was attended by the ED Director, a consultant, a medical officer and a clinical nurse educator. Another by the ED Director, a clinical nurse educator, the Assistant Nursing Unit Manager, and a registered nurse. We estimate an average unit cost of stakeholder attendance based on a typical profile of attendees at stakeholder meetings (see Appendix 5). We then estimate the cost of stakeholder time for each meeting by multiplying the average cost per attendee by the number of attendees at each meeting (assuming one hour per attendee). Total cost of stakeholder time to attend stakeholder meetings across all EDs is estimated as the average cost per attendee multiplied by the total number of attendees across all meetings.
- *Venue hire:* Stakeholder meetings were conducted using meeting rooms adjacent to participating EDs (8 of 8 meetings) and, for some of the meetings that were conducted by teleconference or videoconference, also using Monash University meeting rooms on the Alfred Campus (3 of 8 meetings). We make the conservative assumption that commercial rates would be paid for venue hire and catering for replication or wider roll-out of the NET intervention. Unit costs for meeting room hire are listed in Appendix 5. While these rates reflect advertised prices at a major metropolitan hospital, they are comparable to advertised prices at University (e.g. www.vu.edu.au/convention-centres/room-hire-layout/u-shape) and commercial providers (https://yhotels.com.au/conference-centre/rooms).
- *Consumables:* The majority of stakeholder meetings (5 of 8 meetings) were conducted via teleconference with participants joining the teleconference from their usual place of work. For meetings conducted via teleconference, we apply advertised prices per minute per caller for a large local provider. Two of the remaining three meetings were conducted face-to-face between stakeholders and the NET Project Officer but with the NET investigator attending via Skype. For these meetings, we include venue costs, time and travel costs for the NET Project Officer, and time costs for the NET investigator but no additional costs for telecommunications. For the remaining meeting, the meeting was conducted via videoconferencing. For meetings conducted via videoconference, we apply advertised prices per minute per caller for a large local provider.
- *Travel time & direct costs of travel:* The majority of stakeholder meetings were conducted via teleconference / Skype, with participants joining the teleconference from their usual place of work. Two stakeholder meetings were conducted face-to-face, using hospital meeting rooms adjacent to the participating ED. For face-to-face meetings, the NET Project Officer attended in person necessitating 6 hours of travel-time plus direct costs of travel (air- and taxi-fares) to attend one meeting interstate, and 1.5 hours travel time plus direct costs of travel (taxi-fares) to attend one meeting in metropolitan Melbourne. For all other attendees, meetings took place at their usual place of work and we assume no additional travel time or travel costs associated with attendance. Given that the NET Project Officer travelled as part of her normal work duties during business hours, we cost travel time at the relevant hourly wage rate (see Appendix 5).

**Identifying Local Opinion Leaders:**

Resource-use associated with identifying local opinion leaders can be attributed to personnel and key-informant time.

- *Key informant time:* ED Directors or Senior Consultants were identified as the key-informant for each ED. Key informants were emailed an instruction sheet, specifying characteristics of local opinion leaders and links to supporting references describing the key-informant method. We allow 45 minutes of key informant time to review the instruction sheet, consult with potentially suitable opinion leaders, and return their nominations to the NPO.
- *NET Personnel:* Preparation/distribution of the instruction sheet and development/maintenance of a database of local opinion leaders entailed approximately 3 hours of NPO time and 1 hour of NAO time.

**Train-the-Trainer (TTT) Workshops:** Of the fourteen EDs randomised to the intervention group, thirteen were represented at one or both of the two TTT workshops (each workshop 8 hrs in duration) held in late June (TTT-1, Melbourne) and early August 2014 (TTT-2, Sydney). Resource use associated with delivery of these TTT workshops can be attributed to personnel (NET Project Officer, NET Administration Officer, content experts and NET clinicians), venue hire, local opinion leader time, travel time, and production of materials. Local opinion leaders from one ED were unable to attend either of the TTT workshops and instead attended a shorter meeting (2 hrs duration) with NET project staff held on-site at the relevant ED. While some material delivered as part of TTT workshops/meeting related to the role of local opinion leaders in data collection activities (research costs), there was no clear separation between time/resources devoted to training for research tasks and time/resources devoted to training for intervention delivery. We therefore treat all TTT costs as intervention costs.

- *Local opinion leader time:* TTT-1 and TTT-2 were generally attended by one nursing lead (e.g. Associated Nursing Unit Manager, Clinical Nurse Educator, Registered Nurse) and one medical lead (e.g. ED Director, Consultant, Registrar, Medical Officer) per ED. In one case, the medical lead was unable to attend and we assume that 2 hrs of self-education time was required for the medical lead to familiarise themselves with the TTT materials. In two cases, nursing leads resigned their role in the project after attending TTT-1 and were replaced by another member of the ED’s nursing staff in time for the replacements leads to attend TTT-2. As such, six nursing and six medical leads from six EDs attended TTT-1 whereas TTT-2 was attended by nine nursing and six medical leads from nine EDs. As discussed in Appendix 5, we assume that self-education takes place during downtime at work or after-hours and cost self-education time at the opportunity cost of leisure time ($0 / hour in the base case). While there is some between-ED variation in the role of ED staff selected as local opinion leaders, we make the simplifying assumption that attendance by medical and nursing leads is costed using the unit costs for medical and nursing staff specified in Appendix 5.
- *NET Personnel:* TTT-1 and TTT-2 were facilitated by the NET project staff (x3), NET CIs (x2, and content experts (x4) with backgrounds in neuropsychology, nursing/quality support, and emergency medicine. Each of the facilitators attended TTT-1 and TTT-2 for the entire day and administrative data recorded the same amount of preparation time for all facilitators. Given the lack of variation in delivery and preparation time across facilitators, we calculate total delivery and preparation time across all facilitators and obtain total delivery and preparation cost for each TTT workshop by applying a weighted average of the unit costs for facilitator time given in Appendix 5 to total delivery and preparation time for each workshop.[[1]](#footnote-2) For the TTT meeting held on-site at one ED, NET project staff (x2) facilitated the meeting and so we calculate delivery and preparation cost for the meeting by applying unit costs in Appendix 5 to administrative data re hours of delivery and preparation time for the NET Project Officer and NET Administration Officer.
- *Venue hire:* TTT-1 and TTT-2 were held at the Park Royal Melbourne Airport and Rydges Sydney Airport, respectively. For TTT-1 and TTT-2, administrative records captured actual direct costs of venue hire, audio-visual hire and catering. For the meeting held on-site at one ED, we make the conservative assumption that commercial rates would be paid for venue hire and catering for replication or wider roll-out of the NET intervention. Unit costs for meeting room hire are listed in Appendix 5.
- *Travel time & direct costs of travel:* For TTT-1 and TTT-2, administrative records captured actual direct costs of travel for local opinion leaders by ED and for TTT facilitators. Similarly, administrative records include estimates of travel time to attend the meeting with estimates ranging from ~one hour for Melbourne and Sydney residents to attend workshops in Melbourne and Sydney, and up to 42 hours to make the trip from interstate (including overnight stay). Because travel time and non-travel time away from work were not separately reported, we assume 12 hours of non-travel time away from home whenever an overnight stay was reported. For example, where 24 hours of total travel time (including overnight stay) was reported, we treat 12 hours as travel time and 12 as non-travel time away from home. For non-travel time away from home (overnight stay), we cost attendee time at the opportunity cost of leisure time ($0 / hour in the base case). For travel time, we cost travel time at the 35% of the hourly wage rate under the assumption that mode of travel permitted some measure of productive activity and that travel to TTT workshops was completed either partly or wholly outside of normal work hours (see Appendix 5). For one ED, the TTT workshop was replaced with a meeting held on-site and we assume no additional travel time or travel costs associated with attendance by local leads.
- *Consumables:* Administrative records captured actual direct costs associated with production of materials for delivery of the TTT workshops / meeting. Materials included an intervention manual, CT and PTA assessment tools, and patient information booklets. While materials costs were attributed to each TTT and to the one on-site meeting in administrative records, nearly 75% of the total materials cost was attributed to TTT-1 and notes accompanying data for TTT-2 acknowledged that materials produced for TTT-1 were used in delivery of TTT-2. For calculation of cost at the ED level, we average total materials cost across the 14 intervention group EDs. After inflating the actual direct costs to December 2015 AUD (see Appendix 5), averaging total materials costs across EDs produces a cost per ED of $186; somewhat higher than the $143 recorded in administrative records for the one on-site meeting and somewhat lower than the $313 per ED recorded in administrative records for TTT-1. Administrative records also captured actual direct costs associated with postal distribution of cab-charge vouchers to workshop attendees. After inflating the actual direct costs to December 2015 AUD (see Appendix 5), we attribute these costs to TTT-1 and TTT-2 as recorded in administrative data and then attribute TTT-workshop level costs to EDs based on the number of clinical leads attending each workshop from each ED.[[2]](#footnote-3)

**Local Workshops:** Of the fourteen EDs randomised to the intervention group, thirteen EDs reported local delivery of intervention components over the 7 month period from the early July 2014 to late January 2015. The total reported training time across the thirteen participating EDs was 150.58 hrs in 190 sessions. The average total reported training time across the thirteen participating EDs was 11.6 hrs in an average of 14.6 sessions per ED. For the thirteen ‘participating’ EDs, the number of delivery sessions ranged from five (total reported training time=4.5hrs) to 27 sessions (total reported training time=16 hrs), though another two of the participating EDs had higher total reported training time at 24.5 hrs (20 sessions) and 24 hrs (13 sessions) respectively.

Delivery of local workshops relied on (i) preparation of local opinion leaders to deliver content, (ii) preparation and distribution of supporting tools and materials, (iii) local opinion leader time spent in local delivery, and (iv) ED staff time spent in training and self-education. Costs associated with preparation of local opinion leaders to deliver local workshops have been included in the cost analysis for TTT workshops (see above). All other resource use associated with delivery of local workshops has been included in the cost analysis for local delivery as described below.

- *NET Personnel:* Administrative records included estimates of total NET Admin Officer (NAO) time to: print, compile and pack together boxes with tools and materials (NAO: 30 hours). Administrative records also included estimates of Net Project Officer (NPO) and NAO time associated with customising some tools to local needs including: customising and integrating PTA tools into local pathways (NPO: 1 hr, NAO: 12 hrs), adding logos to materials (NAO: 4 hrs), as well as customising reminder posters and stickers and promotional materials (NAO: 12 hrs). Customising intervention components to local needs would need to be undertaken for each site in any wider roll-out and so we treat associated resource-use as a component of intervention delivery rather than intervention development.[[3]](#footnote-4) Total NET personnel time associated with delivery of local workshops therefore ran to 58 hrs of NET Admin Officer time and one hour of NET Project Officer time.
- *Local opinion leaders:* The total reported time spent in delivery of training sessions across the thirteen participating EDs was 150.58 hrs in 190 sessions (110.75 hrs nurse delivery time + 39.83 hrs doctor delivery time). In addition to time spent in delivery of training sessions, the fidelity data also captured preparation time and time commitment of staff assisting with delivery of each session. The total reported preparation time across the 190 sessions was 98.85 hrs (63.77 hrs nurse preparation time + 35.08 hrs doctor preparation time). The total reported helper time across the 190 sessions was 8.42 hrs (6.67 hrs nurse helper time + 1.75 hrs doctor helper time). Total reported preparation, delivery and helper time across the 190 sessions is therefore 257.85 hours (181.18 hrs nurse time + 76.67 hrs doctor time). While there is some between-ED variation in the role and hourly wage rates of ED staff selected as local opinion leaders, we make the simplifying assumption that preparation and delivery of local training sessions is costed using the unit costs for nurse and doctor time specified in Appendix 5 across all EDs.
- *Local staff time:* Fidelity data included detailed information regarding duration, staff attendance by role, intervention components delivered, and materials delivered for each session. Reported doctor and nurse attendance at local delivery sessions averaged 3.47 (range: 0-32) and 4.82 (range: 0-35) per session, with a total of 915 session attendances by nurses and 659 session attendances by doctors across all sessions. Detailed analysis of intervention fidelity data will be reported elsewhere. For some sessions, fidelity data also included notes describing the scheduling of local delivery session around other ED activities. Very often, these notes suggested that local sessions were opportunistic and that ED staff attended local training sessions during downtime or during routine meetings (e.g. “spoke to staff during nightshift quiet time”, “staff are only able to attend education sessions if they are being covered on the floor”, “…capture(d) staff when I could and gave them a face to face education session”, “brief presentation to consultant ED physician group at the weekly consultant meeting”, “RMO group teaching session hijacked briefly”). Given these comments, we think it unreasonable to attribute person-hours of ED staff attendance to the intervention. Instead, we assume that ED staff attendance occurred entirely during downtime and/or routine meetings and entailed no opportunity cost. Similarly, we assume that self-education takes place during downtime and cost self-education time at the opportunity cost of leisure time ($0 / hour in the base case).
- *Venue hire:* For many sessions, fidelity data also included notes describing the venue for local delivery session or noting that sessions were delivered during routine meetings (e.g. “…telling consultants on floor about project and handing out information about it”, “inservice to take nurses on floor through project …not able to show slides”, “opportunistic education on the ED floor”, “NET was an agenda item in a ward meeting”, “captive audience… ED Registrar teaching session”). Given these comments, we assume that use of seminar/meeting rooms and ED floor space for local delivery of the intervention entailed zero opportunity cost.
- *Consumables:* Each ED was provided with tools and materials designed to reinforce other intervention components (e.g. NET-branded promotional pens, notepads, and mint lollies) and to facilitate recommended management of patients (e.g. PTA tools, CT tools, patient information booklets in English and translated into Arabic, Chinese, Greek, Italian and Vietnamese). Administrative records captured actual direct costs associated with production and distribution of tools and materials. After inflating the actual direct costs to December 2015 AUD (see Appendix 5), we include consumables costs in calculating the total cost of the NET intervention (see Table A1-1 below).

**Table A1-1: Summary of resource-based costing for** delivery of the NET intervention, 2016 AUD

| **Input** | | **Number**  **(A)** | | **Unit cost**  **(B)** | **Total cost**  **(A x B)** |
| --- | --- | --- | --- | --- | --- |
| **Administration / coordination** | | | | | |
|  | NET Administration Officer | HEW 5, Step 5 at 0.3 over 10 weeks ((0.3*10)/48=0.063 EFT) | | $86,557 / EFT | $5,453.10 |
|  | NET Project Officer | Level B, Step 3 at 0.2 over 10 weeks ((0.2*10)/48=0.042 EFT) | | $129,365 / EFT | $5,433.33 |
|  | Office space | 12m^2^ for 0.3 EFT over 10 weeks (12x0.30x10/48=0.75m^2^) | | $233.68/m^2^ | $175.26 |
| ***Sub-total*** | | | | | ***$ 11,061.69*** |
| **Access to the CPG** | | | | | |
|  | Via TTT & Local Workshops | | Attributed to specific intervention components | N/A | N/A |
| ***Sub-total*** | | | | | ***N/A*** |
| **Stakeholder meetings** | | | | | |
|  | Meeting facilitators | 8 meetings, 2 facilitators per meeting = 16 facilitator-sessions | | $172.32 | $2,757.12 |
|  | Stakeholder attendance | 30 attendees, 1 hour per attendee | | $108.21 | $3,246.30 |
|  | Venue costs | 11 meeting rooms, 1 hour per meeting | | $110 per meeting per hour | $1,210.00 |
|  | Videoconferencing | 6 participants, 1 hour per participant = 360 participant-minutes | | $0.09 | $32.40 |
|  | Teleconferencing | 26 participants, 1 hour per participant = 1560 participant-minutes | | $0.08 | $124.80 |
|  | Direct travel costs | Taxis to/from Melbourne airport (including tolls) | | $65 each way | $130.00 |
|  |  | 1 x economy flights Melbourne to interstate airport (return) | | $135 each way | $270.00 |
|  |  | Taxis interstate airport to interstate ED return | | $65 each way | $130.00 |
|  |  | Taxis to/from local ED (including tolls) | | $75 each way | $150.00 |
|  | Travel time | NET Project Officer travel to interstate ED, 6 hrs of 40 hr wk in a 48 wk yr=0.003 EFT | | $129,365 / EFT | $388.10 |
|  |  | NET Project Officer travel to local ED, 1.5 hrs of 40 hr wk in a 48 wk yr=0.001 EFT | | $129,365 / EFT | $101.07 |
| ***Sub-total*** | | | | | ***$8,539.79*** |
| **Identifying local opinion leaders** | | | | | |
|  | Personnel | NET Project Officer, design of instruction sheet ~3 hours | | $67.38 / hour | $202.14 |
|  |  | NET Admin Officer, distribute instruction sheet ~1 hour | | $45.08 / hour | $45.08 |
|  | Key informant time | 45 mins per ED across 14 EDs = 10.5 hours | | $190.30 / hour | $1,998.15 |
| ***Sub-total*** | | | | | ***$2,245.37*** |
| **Train-the-trainer workshops** | | | | | |
|  | Local opinion leader time | (12 x medical leads x 8.5 hrs at TTT-1 & TTT-2) + (1 x medical lead x 2 hrs for one ED-based TTT meeting) = 104 hrs | | $99.40 / hour | $10,337.60 |
|  |  | (15 x nursing leads x 8.5 hrs at TTT-1 & TTT-2) + (1 x nursing lead x 2 hrs for one ED-based TTT meeting) = 129.5 hrs | | $43.76 / hour | $5,666.92 |
|  |  | 1 x medical lead x 2 hrs self-education time = 2 hrs | | $0.00 / hour | $0.00 |
|  | Facilitator time | TTT-1: 76.5 total delivery time plus 20 hours total preparation time = 96.5 hrs | | $82.80 / hour | $7,990.20 |
|  |  | TTT-2: 76.5 total delivery time plus 20 hours total preparation time = 96.5 hrs | | $82.80 / hour | $7,990.20 |
|  |  | ED-based TTT meeting: 9 hrs Project Officer time plus 2 hrs Admin Officer time | | 9*($67.38 / hour) +2*($45.08 / hour) | $696.58 |
|  | Direct travel costs for local opinion leaders (includes flights, taxis and accommodation (where applicable) | TTT-1 (calculated at the ED level but reported at TTT level in line with consents) | | $5499.82 | $5499.82 |
|  |  | TTT-2 (calculated at the ED level but reported at TTT level in line with consents) | | $8349.04 | $8349.04 |
|  | Travel time (excluding non-travel time away from home) costs for local opinion leaders | TTT-1 (calculated at the ED level but reported at TTT level in line with consents) | | $2742.21 | $2742.21 |
|  |  | TTT-2 (calculated at the ED level but reported at TTT level in line with consents) | | $2889.45 | $2889.45 |
|  | Direct travel costs for NET project staff (includes flights, taxis and accommodation (where applicable) | TTT-1, Melbourne | | $1,770.84 | $1,770.84 |
|  |  | TTT-2, Sydney | | $3,905.06 | $3,905.06 |
|  |  | Additional ED-based  TTT meeting | | $40.98 | $40.98 |
|  | Travel time (excluding non-travel time away from home) costs for TTT facilitators | TTT-1, Melbourne (27.5 hrs total facilitator travel time) | | 0.35*($82.80 / hour) | $796.95 |
|  |  | TTT-2, Sydney (46 hrs total facilitator travel time) | | 0.35*($82.80 / hour) | $1,333.08 |
|  |  | Additional ED-based  TTT meeting (5 hrs Project Officer time) | | 0.35*($67.38 / hour) | $117.92 |
|  | TTT Workshop / Meeting venues | TTT-1 venue, Melbourne Airport (9.5 hours) | | $2,953.11 | $2,953.11 |
|  |  | TTT-2 venue, Sydney Airport (9.5 hours) | | $3,787.35 | $3,787.35 |
|  |  | Additional ED-based TTT meeting (4.5 hrs) | | $50.00 set-up fee  $80.00 / hour | $410.00 |
|  | Consumables | Materials including intervention manuals & USBs, 14 EDs | | $185.70 / ED | $2,599.86 |
|  |  | Postage for distributing cab-vouchers to attendees, TTT-1 | | $71.65 | $71.65 |
|  |  | Postage for distributing cab-vouchers to attendees, TTT-2 | | $102.36 | $102.36 |
| ***Sub-total*** | | | | | ***$70,051.18*** |
| **Local Workshops** | | | | | |
|  | Personnel | NET Project Officer: 1 hr | | $67.38 / hour | $67.38 |
|  |  | NET Admin Officer: 58 hrs | | $45.08 / hour | $2,498.64 |
|  | Preparation, delivery and helper time for delivery of local training sessions | Medical lead/helper time: 76.67 hrs | | $99.40 / hour | $7,620.67 |
|  |  | Nurse lead/helper time: 181.18 hrs | | $43.76 / hour | $7,928.58 |
|  | ED staff time attending training sessions | Average training time per session: 47.5 mins. Total attendance, all sessions: 659 doctors, 915 nurses | | $0.00 | $0.00 |
|  | Venue hire | Average training time per session: 47.5 mins. Total sessions: 190 | | $0.00 | $0.00 |
|  | Consumables | PTA tools, printing | | $475.98 | $475.98 |
|  |  | CT tools printing / production | | $2,907.04 | $2,907.04 |
|  |  | Patient information booklets, translation & printing | | $5,087.33 | $5,087.33 |
|  |  | NET-branded promotional materials | | $4,442.46 | $4,442.46 |
|  |  | Postage / courier for tools / materials to EDs | | $1,197.62 | $1,197.62 |
| ***Sub-total*** | | | | | ***$32,225.70*** |
| **Grand Total** | | | | | **$124,123.73** |

1. Here, weights in our weighted average are just the number of each type of investigator. (Cost_Project_Officer*1 + Cost_Admin_Officer*2 + Cost_CI*2 + Cost_Clinical_Expert*3 + Cost_Nursing_Expert*1)/9 = (67.38*1 + 45.08*2 + 122.86*2 + 99.40*3 + 43.76*1)/9 = $82.80 / hour. [↑](#footnote-ref-2)
2. For example, two of the 12 medical/nursing leads attending TTT-1 and one of the 15 leads attending TTT-2 came from the same interstate ED. Postage costs for this ED were therefore calculated as 2/12*(PostageCost_TTT-1) + 1/15*(PostageCost_TTT_2) = $16.70 [↑](#footnote-ref-3)
3. Administrative records also included estimates of NPO and NAO time associated with development of the local workshop component of the NET intervention including: design of PTA tools; design of CT tools including information sheets, pocket cards, and retractable key-rings; design of posters; and design of promotional materials. The analysis presented here is designed to inform decisions regarding wider roll-out of the NET intervention (repeated delivery of the intervention *ex post* of development of the intervention). We therefore exclude intervention development costs from the cost analysis. [↑](#footnote-ref-4)
