# Supplementary material for: Economic evaluation of the NET intervention versus guideline dissemination for management of mild head injury in hospital emergency departments
Source: Implement Sci. 2018 Dec 5;13:147. doi: 10.1186/s13012-018-0834-6 (PMC6280545; doi:10.1186/s13012-018-0834-6)
Supplement: Supplementary file 2 — Appendix 2 - Cost analysis for delivery of the comparator intervention. (DOC 98 kb) [file 13012_2018_834_MOESM2_ESM.doc]

**Appendix 2 - Cost analysis for delivery of the comparator intervention**

Table A2-1 summarises the resource-based costing for delivery of the control condition. Seventeen EDs received the control condition. For control group EDs, 403 medical staff and 548 nursing staff participated in collection of clinician level data but this is a subset of clinicians exposed to the control condition. Resource-use associated with delivery of the control condition can be attributed to: administration and coordination of the control condition, resource-use associated with access to the guideline, resource-use associated with onward dissemination/implementation of the guideline, and education/materials designed to improve data quality. Costs associated with development and standard dissemination of the CPG outside of the NET trial are common and invariant to both treatment and control groups and, on this basis, have not been explicitly included in the cost analysis reported here.

Clinical leads working within EDs randomized to the *control group* were emailed an electronic link to the [*Initial Management of Closed Head Injury in Adults Guideline*](http://www.aci.health.nsw.gov.au/__data/assets/pdf_file/0003/195150/Closed_Head_Injury_CPG_2nd_Ed_Full_document.pdf) and advised to ‘do whatever they would normally do when they became aware of a guideline’. Resource-use associated with dissemination of the guideline to control group EDs is therefore limited to the NET Administration Officer’s time (plus overheads) in compiling mailing lists and drafting emails for the seventeen control group EDs.

Resource-use associated with onward dissemination and active implementation activities within control group EDs is excluded from the cost-analysis reported on the basis that (i) associated costs are likely to be small in magnitude[[1]](#footnote-2), and (ii) an underestimate of control group cost results in an overestimate of the ICER and an underestimate of the NET intervention’s cost-effectiveness. In other words, excluding resource-use associated with onward dissemination and implementation in the control group will yield conservative (downward biased) estimates of cost-effectiveness and this downward bias is likely to be small in magnitude.

In addition to access to the guideline, control group and intervention group EDs also received reminder stickers (10 sheets per ED), posters (10 per ED) and educational sessions (20 mins per ED with either a NET investigator or the NET project officer via telephone). While these resources were intended to improve the quality of administrative data regarding management of mild head injury and could reasonably be described research costs, they may have acted as a reminder of guideline recommendations (and, for intervention group EDs, a reinforcer of other intervention components). Nonetheless, we make no adjustment for resource use associated with measures to improve data quality because: (i) associated costs are likely to be small in magnitude, (ii) any effect attributable to provision of these resources is likely to be small in magnitude, and (iii) costs associated with reminders and education are common and invariant to both treatment and control groups.

Resource-use associated with administration / coordination the control condition entailed a small amount of the NET Administrative Officer’s time (plus overheads) in delivering each component of the control condition (described above). Based on administrative records (e.g. email archives), we estimate that administration/coordination of the control condition entailed no more than 2.5 days of the NET Administration Officer’s time and make allowance for salary (0.5 EFT over one week of a 48 week working year at HEW 5, step 5) and non-research overheads (i.e. office space) on this basis.

**Table A2-1 - Summary of resource-based costing for** delivery of the control condition, 2016 AUD

| **Input** | | **Number**  **(A)** | **Unit cost**  **(B)** | **Total cost**  **(A x B)** |
| --- | --- | --- | --- | --- |
| Access to the CPG | | | | |
|  | NET Administration Officer | HEW 5, Step 5 at 0.5 over 1 week (0.5/48=0.01 EFT) | $86,557 / EFT | $865.57 |
|  | Office space | 12m^2^ for 0.5 over 1 week (12x0.5x1/48=0.12m^2^) | $233.68/m^2^ | $28.05 |
| **Total** | |  |  | **$837.52** |

1. For example, resource-use associated with onward dissemination of the guideline may entail significant self-education time but much of this would occur during downtime at work or after-hours such that self-education time can be costed at the opportunity cost of leisure time ($0 per hour in the base-case). [↑](#footnote-ref-2)
