# Supplementary material for: Economic evaluation of the NET intervention versus guideline dissemination for management of mild head injury in hospital emergency departments
Source: Implement Sci. 2018 Dec 5;13:147. doi: 10.1186/s13012-018-0834-6 (PMC6280545; doi:10.1186/s13012-018-0834-6)
Supplement: Supplementary file 3 — Appendix 3 - Chart audit health service utilisation items. (DOC 137 kb) [file 13012_2018_834_MOESM3_ESM.doc]

# Appendix 3 - Chart audit health service utilisation items

| **FORM 2 : MEDICAL RECORD (Information visible to researchers named on ethics application)** | | | | | | | | | | | | | | | | | | | |
| --- | --- | --- | --- | --- | --- | --- | --- | --- | --- | --- | --- | --- | --- | --- | --- | --- | --- | --- | --- |
| … | | | | | | | | | | | | | | | | | | | |
| **IMAGING OF THE HEAD (ORDERED BY ED STAFF DURING INITIAL ED PRESENTATION**  (Please look up imaging request slip / imaging reporting info) | | | | | | | | | | | | | | | | | | | |
| 5.1 Imaging of the head provided? | ☐ Yes | | | | | 5.1.1 if yes, Reason stated for imaging request ……………………………………………………………………………………………………………………………………………………………………………………………………… | | | | | | | | | | | | | |
|  | ☐ No | | | | |  |  |  |  |  |  |  |  |  |  |  |  |  |  |
| Tick **ALL** that apply | Day | | | | | | | | | | | Time (e.g. 22:10) | | | |  | | | |
| 5.1.2 ☐ CT head | ☐☐/☐☐☐/☐☐☐☐ (DD-MMM-YYYY) | | | | | | | | | | | ☐☐:☐☐ | | | | Details (e.g. CT B/Cx spine/Tx spine) | | | |
| 5.1.3 ☐ Skull X-ray | ☐☐/☐☐☐/☐☐☐☐ (DD-MMM-YYYY) | | | | | | | | | | | ☐☐:☐☐ | | | | Details | | | |
| 5.1.4 ☐ MRI head | ☐☐/☐☐☐/☐☐☐☐ (DD-MMM-YYYY) | | | | | | | | | | | ☐☐:☐☐ | | | | Details | | | |
| 5.1.5 ☐ Other (eg repeat CT) | ☐☐/☐☐☐/☐☐☐☐ (DD-MMM-YYYY) | | | | | | | | | | | ☐☐:☐☐ | | | | Details | | | |
|  | ☐☐/☐☐☐/☐☐☐☐ (DD-MMM-YYYY) | | | | | | | | | | | ☐☐:☐☐ | | | | Details | | | |
|  | ☐☐/☐☐☐/☐☐☐☐ (DD-MMM-YYYY) | | | | | | | | | | | ☐☐:☐☐ | | | | Details | | | |
| 5.1.8 Brain Imaging results: | ☐ Normal | | |  | | | | | | | | | | | | | | | |
|  | ☐ Abnormal | | | ☐ | | | Neurosurgically significant brain injury defined as lesions that will require neurosurgical intervention such as craniotomy, elevation of skull fracture, intracranial pressure monitoring or intubation for head injury or death within 7 days secondary to head injury. Includes acute extradural haematomas, subdural haematomas, subarachnoid haematomas and depressed skull fractures. | | | | | | | | | | | | |
|  |  | | | ☐ | | | Clinically important brain injury: “any acute brain finding revealed on CT that would normally require admission to hospital and neurosurgical follow-up”. All brain injuries should be considered clinically important unless the patient is neurologically intact and has one of the following lesions on CT:  - solitary contusion <5mm diameter  - localised subarachnoid blood < 1mm thick  - smear subdural haematoma <4mm thick  - isolated pneumocephaly  - closed depressed skull # not through the inner table | | | | | | | | | | | | |
|  |  |  |  | ☐ | | | Other | | | | | | | | | | | | |
|  | ☐Unclear / NR | | | If unclear: details ……………………………………………………  ………………………………………………………………………… | | | | | | | | | | | | | | | |
| **DISCHARGE HOME FROM ED / ADMISSION TO WARD (PLEASE NOTE: SSU = ED)** | | | | | | | | | | | | | | | | | | | |
| 6.1 Discharge date / time | | ☐☐/☐☐☐/☐☐☐☐ (DD-MMM-YYYY) ☐☐:☐☐(HH-NN; eg 23:1) | | | | | | | | | | | | | | | | | |
| 6.2 Destination | | ☐ Home | | | ☐ Ward | | | ☐ Neurosurgical intervention | | | | | | | | | ☐ Unclear / NR | | |
| If admitted to ward: | | Reason…………………………………………………………………………………… | | | | | | | | | | | | | | | | | |
| If unclear: | | Details..…………………………………………………………………………………… | | | | | | | | | | | | | | | | | |
| 6.3 Referral to GP for follow-up | | ☐ Yes | | | | If stated: referred to GP OR you find letter addressed to the GP saying “pls review this patient” | | | | | | | | | | | | | |
|  |  | ☐ No | | | | If stated: no need to be seen by GP | | | | | | | | | | | | | |
|  |  | ☐ Unclear / NR | | | | If unclear: provide details …………………………………..  …………………………………………………………………………………………………………………………………… | | | | | | | | | | | | | |
| 6.4 Discharge summary provided for local doctor | | ☐ Yes | | | | If stated: Discharge summary provided OR letter / carbon-print found | | | | | | | | | | | | | |
|  |  | ☐ No | | | | If noted that no discharge summary provided | | | | | | | | | | | | | |
|  |  | ☐ Unclear / NR | | | | NR: no letter / carbon-print found  If unclear: details……………………………………………..  ………………………………………………………………….…………………………………………….…………………… | | | | | | | | | | | | | |
| 6.5 Head injury advice given to patient: | | ☐ Yes | | | | If recorded: “advice provided, or you find any evidence of advice provided” | | | | | | | | | | | | | |
|  |  |  |  |  |  | If YES: mode: | | | | | ☐ Written | | | ☐ Verbal | ☐ Both | | | | ☐ Unclear |
|  |  | Notes | | | | eg regarding what advice consisted of …………………………………………………………………………………………………………………………………… | | | | | | | | | | | | | |
|  |  | ☐ No | | | | If noted that no advice was provided | | | | | | | | | | | | | |
|  |  | ☐ Unclear / NR | | | | If unclear: details …………………………………………………………………  …………………………………………………………………  ………………………………………………………………… | | | | | | | | | | | | | |
| **REPRESENTATION** | | | | | | | | | | | | | | | | | | | |
| 7.1 Did the patient represent **within a month?** | | ☐ Yes | 7.1.1 If so, was the visit in relation to their head injury or for any other reason | | | | | | | | | | | | | | | | |
|  |  |  | ☐ Head injury  details……………………………………………………………………………………… | | | | | | | | | | ☐ Other reason  details……………………………………………………………………………………… | | | | ☐ Unclear: details  …………………………………………………………………………………… | | |
|  |  | ☐ No |  | | | | | | | | | | | | | | | | |
| **INPATIENT CODING [only complete for patients who have been admitted to ward]** | | | | | | | | | | | | | | | | | | | |
| 8.1 DRG available: | | ☐ Yes | | | | | | | | If YES: code: ……………………………………………………… | | | | | | | | | |
|  |  | ☐ No | | | | | | | | If NO: please list ALL icd-10 (or equivalent) codes relevant to inpatient stay: ………………………………………………………………………………………….…………………………………………………………………………………………………………………………………… | | | | | | | | | |
| **HEAD INJURY RELATED TREATMENT (INITIAL ED PRESENTATION OR WARD OR IN ED DURING REPRESENTATION)** please collect information on potential treatment elements: | | | | | | | | | | | | | | | | | | | |
| 8.2 Hyperventilation | | ☐ Yes | | | | | | | ☐ No | | | | | | | | |  | |
| 8.3 Mannitol | | ☐ Yes | | | | | | | ☐ No | | | | | | | | |  | |
| 8.4 Surgical decompression | | ☐ Yes | | | | | | | ☐ No | | | | | | | | |  | |
| 8.5 Anti-convulsants | | ☐ Yes | | | | | | | ☐ No | | | | | | | | |  | |
| 8.6 ICP monitoring | | ☐ Yes | | | | | | | ☐ No | | | | | | | | |  | |
| 8.7 CT scan **(ward or representation ONLY)** | | ☐ Yes | | | | | | | ☐ No | | | | | | | | | ☐ N/A | |
| 8.8 MRI **(ward or representation ONLY)** | | ☐ Yes | | | | | | | ☐ No | | | | | | | | | ☐ N/A | |
| 8.9 ICU admission | | ☐ Yes | | | | | | | ☐ No | | | | | | | | |  | |
| 8.10 Analgesia | | ☐ Yes | | | | | | | ☐ No | | | | | | | | |  | |
| 8.11 Other (eg sedation, intubation, neurosurgical consult, or retrieval consult) | |  | | | | | | | | | | | | | | | | | |
