# Supplementary material for: Economic evaluation of the NET intervention versus guideline dissemination for management of mild head injury in hospital emergency departments
Source: Implement Sci. 2018 Dec 5;13:147. doi: 10.1186/s13012-018-0834-6 (PMC6280545; doi:10.1186/s13012-018-0834-6)
Supplement: Supplementary file 4 — Appendix 4 - Post-discharge health service utilisation items. (DOC 105 kb) [file 13012_2018_834_MOESM4_ESM.doc]

# Appendix 4 - Post-discharge health service utilisation items

[Health Service Utilisation]

The following questions are in relation to the use of healthcare facilities in the last **FOUR** weeks.

5.1 Did you visit the emergency department after your first visit in relation to your head injury? (Yes / No)

5.1.1 If yes, was that because of the head injury or for any other reason?

[…] because of the head injury

[…] because of another reason

[…] both

5.2 Did you visit other healthcare providers in the last **FOUR** weeks, for example your GP, head injury clinic, neurologist etc, because of your head injury? (Yes / No)

5.2.1 If yes, how many visits did you make to each?

[Type: ………………………………………..] [Number of visits: …………]

[Type: ………………………………………..] [Number of visits: …………]

[Type: ………………………………………..] [Number of visits: …………]

[Type: ………………………………………..] [Number of visits: …………]

5.3 In the last 4 weeks, have you taken any drugs for head ache? [Yes / No]

[If yes, PER DRUG for up to four drugs]:

1. What medication was that? [name & dose?]

2. How many times do you take it per day?

3. How many days in the past four weeks did you take it?

| Name (brand / generic) | Dose (e.g. 200 mg) | Times per day | Duration use  (….days out of last 4 weeks) |
| --- | --- | --- | --- |
|  |  |  |  |
|  |  |  |  |
|  |  |  |  |
|  |  |  |  |

5.4 In the last 4 weeks, have you taken any drugs to make you feel less anxious? [Yes / No]

[If yes, PER DRUG for up to four drugs]:

1. What medication was that? [name & dose?]

2. How many times do you take it per day?

3. How many days in the past four weeks did you take it?

| Name (brand / generic) | Dose (e.g. 200 mg) | Times per day | Duration use  (….days out of last 4 weeks) |
| --- | --- | --- | --- |
|  |  |  |  |
|  |  |  |  |
|  |  |  |  |
|  |  |  |  |

5.5 In the last 4 weeks, have you taken any drugs to help you sleep better? [Yes / No]

[If yes, PER DRUG for up to four drugs]:

1. What medication was that? [name & dose?]

2. How many times do you take it per day?

3. How many days in the past four weeks did you take it?

| Name (brand / generic) | Dose (e.g. 200 mg) | Times per day | Duration use  (….days out of last 4 weeks) |
| --- | --- | --- | --- |
|  |  |  |  |
|  |  |  |  |
|  |  |  |  |
|  |  |  |  |

5.6 In the last 4 weeks, have you taken any drugs for nausea? [Yes / No]

[If yes, PER DRUG for up to four drugs]:

1. What medication was that? [name & dose?]

2. How many times do you take it per day?

3. How many days in the past four weeks did you take it?

| Name (brand / generic) | Dose (e.g. 200 mg) | Times per day | Duration use  (….days out of last 4 weeks) |
| --- | --- | --- | --- |
|  |  |  |  |
|  |  |  |  |
|  |  |  |  |
|  |  |  |  |
