# Supplementary material for: Economic evaluation of the NET intervention versus guideline dissemination for management of mild head injury in hospital emergency departments
Source: Implement Sci. 2018 Dec 5;13:147. doi: 10.1186/s13012-018-0834-6 (PMC6280545; doi:10.1186/s13012-018-0834-6)
Supplement: Supplementary file 5 — Appendix 5 - Unit costs by category of resource use. (DOC 235 kb) [file 13012_2018_834_MOESM5_ESM.doc]

**Appendix 5 -** **Unit costs by category of resource use**

Table A5-1: Delivery of the intervention and control conditions

| **Category/Item** | **Description** | **Unit cost** | **Source** |
| --- | --- | --- | --- |
| **Overheads** | | | |
| Office space | Rental charge per year per m^2^ for office-space and utilities on the Monash Medical Centre Campus. | $233.68 / per year per m^2^ | 2016 Monash University Direct Facilities Charges |
| **Personnel** | | | |
| Investigator time | Level E, Step 1 annual salary of $169,027 plus 39.56% salary on-costs | $235,894 / year | 2016 Monash Academic Salary Scale |
| Project Officer | Level B, Step 3 annual salary of $98,971 plus 30.71% salary on-costs | $129,365 / year |  |
| Admin Officer | HEW 5, Step 5 annual salary of $66,221 plus 30.71% salary on-costs | $86,557 / year | 2016 Monash Professional Salary Scale |
| Stakeholder MTG facilitator | Basic lecture (2 hours preparation/training time per 1 hour of delivery) | $177.49 / hour of delivery | 2016 Monash Sessional Academic Rates |
| Stakeholder attendance at meetings | ED Director (HN64: hourly rate for Executive Fractional Specialist at Top of Range working 17.6+ hrs/week) | $190.30 / hour | AMA Victoria Rates of Pay for Specialists in Public Hospitals |
|  | ED Consultant (HN39: hourly rate for Fractional Specialist Year 5 working 17.6+ hrs/week) | $145.70 / hour | AMA Victoria Rates of Pay for Specialists in Public Hospitals |
|  | ED Medical Officer (HM16: hourly rate for Medical Officer Year 3) | $53.09 / hour | AMA Victoria Rates of Pay for Doctors in Training |
|  | ED Clinical Nurse Educator (Registered Nurse Grade 4B weekly ordinary full-time wage of $1,662.90 per 38 hour week) | $43.76 / hour | Nurses and Midwives (Victorian Public Sector) (Single Interest Employers) Enterprise Agreement 2012-2016 |
| Key informant time | ED Director (HN64: hourly rate for Executive Fractional Specialist at Top of Range working 17.6+ hrs/week) | $190.30 / hour | AMA Victoria Rates of Pay for Specialists in Public Hospitals |
| Local opinion leader attendance at TTT workshops | Medical lead: Average of (HN39: hourly rate for Fractional Specialist Year 5 working 17.6+ hrs/week=$145.70) and (HM16: hourly rate for Medical Officer Year 3=$53.09 / hour) | $99.40 / hour | AMA Victoria Rates of Pay for Specialists in Public Hospitals & AMA Victoria Rates of Pay for Doctors in Training |
|  | Nursing lead (Registered Nurse Grade 4B weekly ordinary full-time wage of $1,662.90 per 38 hour week) | $43.76 / hour | Nurses and Midwives (Victorian Public Sector) (Single Interest Employers) Enterprise Agreement 2012-2016 |
| Facilitator preparation and delivery time for TTT workshops | Investigator: Level E, Step 1 annual salary of $169,027 plus 39.56% salary on-costs | $235,894 / year  $122.86 / hour[[1]](#footnote-2) | 2016 Monash Academic Staff Salary Scale |
|  | Project officer: Level B, Step 3 annual salary of $98,971 plus 30.71% salary on-costs | $129,365 / year  $67.38 / hour |  |
|  | Administration officer: HEW 5, Step 5 annual salary of $66,221 plus 30.71% salary on-costs | $86,557 / year  $45.08 / hour | 2016 Monash Academic Staff Salary Scale |
|  | Clinical experts: Average of (HN39: hourly rate for Fractional Specialist Year 5 working 17.6+ hrs/week=$145.70) and (HM16: hourly rate for Medical Officer Year 3=$53.09 / hour) | $99.40 / hour | AMA Victoria Rates of Pay for Specialists in Public Hospitals & AMA Victoria Rates of Pay for Doctors in Training |
|  | Nursing expert: (Registered Nurse Grade 4B weekly ordinary full-time wage of $1,662.90 per 38 hour week) | $43.76 / hour | Nurses and Midwives (Victorian Public Sector) (Single Interest Employers) Enterprise Agreement 2012-2016 |
| Local opinion leader preparation and delivery of local training sessions | Medical lead: Average of (HN39: hourly rate for Fractional Specialist Year 5 working 17.6+ hrs/week=$145.70) and (HM16: hourly rate for Medical Officer Year 3=$53.09 / hour) | $99.40 / hour | AMA Victoria Rates of Pay for Specialists in Public Hospitals & AMA Victoria Rates of Pay for Doctors in Training |
|  | Nursing lead (Registered Nurse Grade 4B weekly ordinary full-time wage of $1,662.90 per 38 hour week) | $43.76 / hour | Nurses and Midwives (Victorian Public Sector) (Single Interest Employers) Enterprise Agreement 2012-2016 |
| Clinician self-education time | Self-education is assumed to take place during downtime at work or after-hours and assume that self-education time can be costed at the opportunity cost of leisure time. | $0.00 | Drummond et al suggest a value for lost leisure time of zero *in the base case* but concede arguments could also be made for valuing lost leisure time at average wage or average overtime rates. |
| **Venues** | | | |
| Meeting rooms for stakeholder meetings | Small meeting room with a 10 person capacity, major metropolitan area. | $50.00 set-up fee / room  $80.00 / hour  $350.00 / day | RMH Function & Convention Centre, Room Configurations & Fee Structure |
| TTT Workshop / Meeting venues  TTT Workshop / Meeting venues | Actual direct costs for TTT-1 inflated from June quarter 2014 to December quarter 2015 using the all-items Consumer Price Index: Cost*CPI_12/15 / CPI_06/14=$2,885*(108.4/105.9) | $2,953.11 | Administrative records of costs incurred |
|  | Actual direct costs for TTT-2 inflated from June quarter 2014 to December quarter 2015 using the all-items Consumer Price Index: Cost*CPI_12/15 / CPI_06/14=$3,700*(108.4/105.9) | $3,787.35 | Administrative records of costs incurred |
|  | Small meeting room with a 10 person capacity for TTT meeting ED, major metropolitan area. | $50.00 set-up fee / room  $80.00 / hour  $350.00 / day | RMH Function & Convention Centre, Room Configurations & Fee Structure |
| **Travel costs** | | | |
| Direct transport costs for stakeholder meetings | Direct costs of taxi travel estimated based on distance travelled and per km rates plus standard daytime flag-falls | $65.00-$75.00 | Victorian Taxi Fares |
|  | Economy airfare Melbourne to interstate (one-way) | $135.00 | [www.qantas.com](http://www.qantas.com/), accessed 15^th^ April 2016 |
| Direct transport costs for local leads to attend TTT Workshops *(includes taxis, flights and accommodation)* | Actual direct costs for each ED to attend TTT-1 inflated from June quarter 2014 to December quarter 2015 using the all-items Consumer Price Index: Cost*CPI_12/15 / CPI_06/14=$ED_Cost*(108.4/105.9) | $5,499.82 | Administrative records of costs incurred (calculated at ED level but reported at TTT level to comply with consents) |
|  | Actual direct costs for each ED to attend TTT-2 inflated from June quarter 2014 to December quarter 2015 using the all-items Consumer Price Index: Cost*CPI_12/15 / CPI_06/14=$ED_Cost*(108.4/105.9) | $8,349.04 |  |
| Direct transport costs for NET staff / CIs to attend TTT workshops *(includes taxis, flights and accommodation)* | Actual direct costs for TTT-1 inflated from June quarter 2014 to December quarter 2015 using the all-items Consumer Price Index: Cost*CPI_12/15 / CPI_06/14=$1730*(108.4/105.9) | $1,770.84 | Administrative records of costs incurred |
|  | Actual direct costs for TTT-2 inflated from June quarter 2014 to December quarter 2015 using the all-items Consumer Price Index: Cost*CPI_12/15 / CPI_06/14=$3815*(108.4/105.9) | $3,905.06 |  |
|  | Actual direct costs for additional ED-based  TTT meeting inflated from June quarter 2014 to December quarter 2015 using the all-items Consumer Price Index: Cost*CPI_12/15 / CPI_06/14=$40*(108.4/105.9) | $40.98 |  |
| Travel time to TTT workshops | Per hour of travel time completed partly outside of normal business hours and permitting productive activity.[[2]](#footnote-3) | 35% of hourly wage rate | Transportation Cost and Benefit Analysis II - Travel Time Costs |
| **Other consumables** | | | |
| In-house printing of CPG | 2016 advertised retail prices for black and white printing (per side) on 100 GSM paper at Officeworks | $0.10 | Officeworks Product & Service Price Guide |
| Teleconference calls, Stake-holder Meetings | Per caller per minute | $0.08 | Eureka Conferencing, Audio & Video Confercing Rates |
| Videoconference calls, Stake-holder Meetings | Per caller per minute | $0.09 | Eureka Conferencing, Audio & Video Confercing Rates |
| Direct cost of materials for TTT Workshops | Actual direct costs for production of intervention manuals inflated from June 2014 to December 2015 using the all-items Consumer Price Index: Cost*CPI_12/15 / CPI_06/14=$2,540*(108.4/105.9) | $2,599.86 | Administrative records of costs incurred |
| Postage costs for TTT-1 & TTT-2 | Actual direct costs for TTT-1 inflated from June 2014 to December 2015 using the all-items Consumer Price Index: Cost*CPI_12/15 / CPI_06/14=$70*(108.4/105.9) | $71.65 | Administrative records of costs incurred |
|  | Actual direct costs for TTT-2 inflated from June 2014 to December 2015 using the all-items Consumer Price Index: Cost*CPI_12/15 / CPI_06/14=$100*(108.4/105.9) | $102.36 |  |
| Printing, PTA tools using hosp templates as req. | Actual direct costs inflated from June 2014 to December 2015 using the all-items Consumer Price Index: Cost*CPI_12/15 / CPI_06/14=$465*(108.4/105.9) | $475.98 | Administrative records of costs incurred |
| Production / printing, CT tools including pocket cards & key-rings | Actual direct costs inflated from June 2014 to December 2015 using the all-items Consumer Price Index: Cost*CPI_12/15 / CPI_06/14=$2,840*(108.4/105.9) | $2,907.04 | Administrative records of costs incurred |
| Translation & printing, patient info booklets for local delivery | Actual direct costs inflated from June 2014 to December 2015 using the all-items Consumer Price Index: Cost*CPI_12/15 / CPI_06/14=$4,970*(108.4/105.9) | $5,087.33 | Administrative records of costs incurred |
| NET-branded promotional materials | Actual direct costs inflated from June 2014 to December 2015 using the all-items Consumer Price Index: Cost*CPI_12/15 / CPI_06/14=$4,340*(108.4/105.9) | $4,442.46 | Administrative records of costs incurred |
| Postage / courier, materials to EDs for local delivery | Actual direct costs inflated from June 2014 to December 2015 using the all-items Consumer Price Index: Cost*CPI_12/15 / CPI_06/14=$1,170*(108.4/105.9) | $1,197.62 | Administrative records of costs incurred |

**Table A5**-2: Health Services

| **Category / Item** | **Description** | **Unit cost** | **Source** |
| --- | --- | --- | --- |
| ED presentation[[3]](#footnote-4),[[4]](#footnote-5) | URG v1.4 code 10: Admitted T2_Injury (price weight=0.2847) where ATS Category 2 includes ‘severe localised trauma - major fracture’ | 0.2847*$4,971= $1,415.24 | Nationally Efficient Price Determination 2015-16 |
|  | URG v1.4 code 20: Admitted T3 Injury (price weight=0.2012) where ATS Category 3 includes ‘head injury with short LOC- now alert’ | 0.2012*$4,971= $1,000.17 |  |
|  | URG v1.4 code 34: Admitted T4 Injury (price weight=0.1548) where ATS Category 4 includes ‘minor head injury, no loss of consciousness’ | 0.1548* $4,971 =$769.51 |  |
|  | URG v1.4 code 44: Non Admitted T2_Injury (pw=0.1722) where ATS Category 2 includes ‘severe localised trauma - major fracture’ | 0.1548* $4,971 =$856.01 |  |
|  | URG v1.4 code 50: Non Admitted T3 Injury (price weight=0.1207) where ATS Category 3 includes ‘head injury with short LOC- now alert’ | 0.1207*$4,971= $600.00 |  |
|  | URG v1.4 code 58: Non Admitted T4_Injury (price weight=0.0779) where ATS Category 4 includes ‘minor head injury, no loss of consciousness’ | 0.0779*$4,971= $387.24 |  |
| ED presentation less average imaging cost[[5]](#footnote-6) | URG v1.4 code 10: Admitted T2_Injury (price weight=0.2847) less 2.9% average imaging cost | $1,415.24-$41.04=  $1,374.20 | Nationally Efficient Price Determination 2015-16 |
|  | URG v1.4 code 20: Admitted T3 Injury (price weight=0.2012) less 2.9% average imaging cost | $1,000.17-$29.00= $971.17 |  |
|  | URG v1.4 code 34: Admitted T4 Injury (price weight=0.1548) less 2.9% average imaging cost | $769.51-$22.32= $747.19 |  |
|  | URG v1.4 code 44: Non Admitted T2_Injury (pw=0.1722) less 2.9% average imaging cost | $856.01-$24.82= $831.19 |  |
|  | URG v1.4 code 50: Non Admitted T3 Injury (price weight=0.1207) less 2.9% average imaging cost | $600.00-$17.40= $582.60 |  |
|  | URG v1.4 code 58: Non Admitted T4_Injury (price weight=0.0779) less 2.9% average imaging cost | $387.24-$11.23= $376.01 |  |
| Diagnostic imaging | In-hospital DI by type (X-ray, CT, MRI) & body-region (e.g. head, CSpine, Upper Extremity) from chart audit | Scheduled fee | Schedule of Medicare Benefits |
| Time-use for recommended behaviours in ED | PTA assessment using validated screening too & INFO (provision of written information on discharge): Registered Nurse Grade 4B weekly ordinary full-time wage of $1,662.90 per 38 hour week | $43.76 / hour | Nurses and Midwives (Victorian Public Sector) (Single Interest Employers) Enterprise Agreement 2012-2016 |
|  | CT assessment using clinical decision rule or guideline-developed criteria: Average of (HN39: hourly rate for Fractional Specialist Year 5 working 17.6+ hrs/week=$145.70) and (HM16: hourly rate for Medical Officer Year 3=$53.09 / hour) | $99.40 / hour | AMA Victoria Rates of Pay for Specialists in Public Hospitals & AMA Victoria Rates of Pay for Doctors in Training |
| mTBI related medications | Includes analgesia (codeine+paracetamol), anti-convulsants (phenytoin) | DPMQ for relevant PBS items | Schedule of Pharmaceutical Benefits |
| ICU admissions | Intensive care unit adjustment | (0.0440*ICU_hrs)  *$4,971= | Nationally Efficient Price Determination 2015-16 |
| Inpatient admissions | AR-DRG v7.0 (mapped from ICD-10 or SNOMED codes where AR-DRG codes not reported) | pw*NEP=pw*$4,971 | Nationally Efficient Price Determination 2015-16 |
| Outpatient clinic | Tier 2 Clinic V4.0: 20.07 General Surgery Outpatient Clinic (pw=0.0591) | 0.0591*$4,971= $293.79 | Nationally Efficient Price Determination 2015-16 |
| Referral to GP at discharge | Level B attendance at consulting rooms: MBS item 23 | $37.05 | Schedule of Medicare Benefits |
| Re-presentations to ED | URG v1.4 code 58: Non Admitted T4_Injury (price weight=0.0779) where ATS Category 4 includes ‘minor head injury, no loss of consciousness’ | 0.0779*$4,971= $387.24 | Nationally Efficient Price Determination 2015-16 |
| Readmission / scheduled surgery | AR-DRG v7.0: I17B Maxillo-Facial Surgery W/O CC (pw=1.4633) | 1.4633*$4,971=  $7,274.06 | Nationally Efficient Price Determination 2015-16 |
|  | AR-DRG v7.0: I77B Fractures of Pelvis W/O Catastrophic or Severe CC (pw=1.1378) | 1.1378*$4,971=  $5,656.00 |  |
|  | AN-SNAP V3.0 code 3-101 Palliative Care Overnight Palliative Care Palliative care, admit for assessment only (pw=0.3666) | 0.3666*$4,971=  $1,822.37 |  |
|  | AR-DRG v7.0: Z61A Signs and Symptoms (pw=0.9023) | 0.9023**$4,971=  $4,485.33 |  |

1. Assuming 40 hours per week in a 48 week year; total of 1920 hrs per EFT salary. [↑](#footnote-ref-2)
2. The cost of travel time is just the value of the next best alternative use of that time (eg. paid work, voluntary work, leisure). Litman [38] summarised a number of findings with respect to the cost of travel, concluding that personal travel time is usually estimated at one-quarter to one-half of prevailing wage rates, that travel time costs tend to increase in line with income, and that paid travel time should be valued at the opportunity cost of work time. With respect to paid travel time, not all travel time is lost time and - under favourable conditions – travel time entails a lower per-minute cost because passengers can relax or perform productive work. For example, travel as a transit passenger using transport with a high level of comfort, convenience and reliability carries a cost of 35% of prevailing wage rates. In the case of travel to attend the TTT Workshops, travel occurred either outside of business hours on the day of the workshop or on the day prior, typically with some travel occurring within normal business hours and some after normal business hours. For this reason, unit costs for paid travel time are not appropriate and should instead reflect some weighted average of costs for paid and personal travel time. Note, however, that the mode of travel to TTT Workshops (taxi, self-drive, plane travel) permits some measure of productive activity suggesting that travel time should be valued at somewhat less than prevailing wage rates. [↑](#footnote-ref-3)
3. Price of an emergency department or emergency service: ABF Activity = {PW x (1 + AInd)} x NEP where PW is price weight, AInd is indigenous adjustment and NEP is nationally efficient price. Our data does not include indigenous status and so formula reduces to PW*NEP. [↑](#footnote-ref-4)
4. For examples of triage conditions, see Triage in the Emergency Department [41] [↑](#footnote-ref-5)
5. Imaging costs included in calculation of average price weights account for 2.9% of total ED costs. Here, imaging costs are costed based on micro data and so we subtract average imaging cost from cost per ED presentation. See Table 17 in IHPA [43] [↑](#footnote-ref-6)
