# Supplementary material for: Economic evaluation of the NET intervention versus guideline dissemination for management of mild head injury in hospital emergency departments
Source: Implement Sci. 2018 Dec 5;13:147. doi: 10.1186/s13012-018-0834-6 (PMC6280545; doi:10.1186/s13012-018-0834-6)
Supplement: Supplementary file 7 — Appendix 7 - Sensitivity analyses: potential impact of missing data and selection bias. (DOC 106 kb) [file 13012_2018_834_MOESM7_ESM.doc]

**Appendix 7 – Sensitivity analyses: potential impact of missing data & selection bias**

Thirty-one EDs were randomised (17 control EDs and 14 intervention EDs), with all 31 being included in the analysis for our clinical practice outcomes (PTA, INFO and SAFED). From these 31 EDs, a total of 1943 patients were identified from medical records for inclusion in the study; with all 1943 being included in analysis for the primary outcome (PTA). Data on the primary outcome, minimisation factors and pre-specified confounders were complete for all 1943 patients included in the study; largely as a consequence of the inclusion / exclusion criteria that were applied when identifying patients for inclusion in the trial. Specifically, potentially eligible patients were identified from retrospective chart audit of the ED medical records. Data on injury type (included: trauma to head; excluded: penetrating injury / non-traumatic injury) and initial GCS were required in order to assess patients against inclusion / exclusion criteria. In addition, patients with missing medical records were excluded prior to randomisation because assessment of the primary outcome required access to medical records. Detailed reporting of recruitment, randomisation and participant flow can be found in the main trial report (submitted to *Implementation Science* as a companion paper to the economic evaluation paper).

On the cost side of the equation, complete data for the full sample of 1943 patients were available for most cost categories included in the base-case analysis and for all minimisation factors and pre-specified confounders. Missing data for a small number of patients for some cost categories included in the base-case analysis were replaced using assumption or single-imputation as appropriate. Assumptions were specified to err on the side of caution and *avoid* bias in favour of the evaluated intervention. For example, we were unable to code ct_item from imaging_details_ct for n=36 patients (34 from the treatment group and 2 from the control group). In this case we assumed ‘CT Head without contrast’ at a cost of $195.05 per scan where imaging_type_ct='YES' but ct_details missing.

Cost-categories with more significant levels of missingness (i.e. URG and DRG costs) were excluded from the base-case analysis for reasons specified at pp11-12. Replacement of DRG codes missing from patient records entailed a rule-based mapping from ICD-10/SNOMED codes to DRG codes. Replacement of URG codes missing from patient records followed a rule-based mapping based on individual patient data regarding loss of consciousness, skull fracture and discharge destination. Replacement of missing URG codes, DRG codes or both was necessary for all patients in the sample such that a comparison of complete and incomplete cases is not informative. While examining sensitivity to the specifics of rule-based mappings or other methods of imputation may provide a more complete guide of parameter uncertainty for URG and DRG costs, we think the additional complexity introduced by this type of sensitivity analysis would make the paper much more difficult to navigate. Instead, we conducted sensitivity analyses to evaluate the impact of inclusion / exclusion of URG and DRG costs calculated as described above and in the manuscript.

For secondary clinical practice outcomes in the NET sample and health outcomes in the NET-Plus sample, missingness relates to the outcomes of interest rather than covariates. However, the mechanism for this missingness is different for secondary clinical practice outcomes in the NET sample than for secondary health outcomes in the NET-Plus sample. For health outcomes in the NET-Plus sample, a subset of trial EDs participated in the NET-Plus component of the trial (14 control EDs and 10 intervention EDs). Patients treated in NET-Plus EDs who were included in the chart audit for the NET component of the trial were then invited to participate in health outcomes data collection via telephone interviews. A total of 218 control and 125 intervention participants were included in the NET-Plus sample via this process, all of which were included in analysis for the Rivermead Post-concussion Symptoms Scale and HADS Anxiety Scale. For the SF6D, SF-12 data (from which the SF6D was calculated) were not recorded for 10 of the 218 control group participants and 2 of the 125 intervention group participants.

For secondary clinical practice outcomes in the NET sample, INFO and SAFED were only assessable in a subset of patients. INFO was only assessable in the subset of patients for whom information provision was recommended upon discharge from the ED (n=944 control group patients and 785 intervention group patients). This excluded patients who were not discharged home (patients who were admitted to the ward, admitted for neurosurgical intervention or transferred to another facility). For SAFED, assessment required information on three clinical practice outcomes: PTA, INFO and CT, where CT denoted whether a CT scan was provided in the presence of a risk factor that justifies the scan (age 65 or older; GCS<15; amnesia; suspected skull fracture; vomiting and coagulopathy). CT was therefore only assessed in the cohort of patients for whom risk criteria were recorded and so SAFED could only be constructed for this subset of the full sample.

Where missingness relates to the outcomes of interest rather than covariates (as is the case for all secondary outcomes in the present study), multiple imputation cannot improve upon the complete case analysis unless we can include auxiliary predictors for the outcome of interest in the MI model that have been excluded from the analysis model (see, for example, Von Hippel, 2007). For the present paper, we *do* have access to auxiliary predictors due to the availability of multiple outcomes (e.g. we have complete data for our primary outcome and this could be included in MI analysis to replace missing values for secondary outcomes). The inclusion of multiple outcomes in the MI model may, however, allow estimated treatment effects for secondary outcomes to ‘borrow strength’ from estimated treatment effects for the primary outcome. To evaluate the potential impact of selection on complete case analyses for secondary outcomes, we therefore take a different approach and evaluate sensitivity of estimates for our primary outcome (PTA) to the same selection mechanisms that result in missingness for each of our secondary outcomes. Table A below compares estimated treatment effects for PTA in the full sample against estimated treatment effects for PTA in the subsamples used for analysis of secondary outcomes.

**Table A7-1: Effect of the intervention on the primary outcome (PTA), sensitivity to sample selection**

| **Variable; sample** | **No. patients (EDs)** | | **N (%) / Mean (SE)** | | **Increment, raw (95%CI)**^ | **Increment, adjusted (95%CI)^‡^** |
| --- | --- | --- | --- | --- | --- | --- |
|  | **Rx** | **Control** | **Rx** | **Control** |  |  |
| ***PTA*** | | | | | | |
| Full NET sample | 893 (14) | 1050 (17) | 117 (13.1%) | 12 (1.1%) | 11.96% (9.8, 14.1) | 13.63% (8.3, 19.0) |
| INFO sample | 785 (14) | 944 (17) | 115 (14.6%) | 12 (1.3%) | 13.38% (11.0, 15.8) | 14.82% (8.8, 20.9) |
| SAFED sample | 402 (14) | 413 (17) | 64 (15.9%) | 9 (2.2%) | 13.74% (9.9, 17.6) | 16.22% (8.7, 23.7) |
| Full NET-Plus sample | 125 (10) | 218 (14) | 19 (15.2%) | 1 (0.5%) | 14.74% (9.8, 19.7) | 13.69% (5.2, 22.2) |
| HRQoL sample | 123 (10) | 208 (14) | 19 (15.2%) | 1 (0.5%) | 14.97% (9.8, 19.7) | 13.69% (5.2, 22.2) |

While different samples give different estimates, results reported in Table A7-1 suggest that estimated treatment effects for the primary outcome are inflated (upward bias) and less precise when estimated in the subsamples used for analysis of secondary outcomes (as compared to estimates obtained from analysis of the full sample). Given the lack of any significant difference between treatment and control groups on secondary outcomes (see Table 3 of the main manuscript), selection and missingness would need to have exerted a downward bias on estimated treatment effects for secondary outcomes in order for removal of any such bias to change our conclusions. The results reported in Table A suggest that the reverse is the case (selection and missingness appear to be exerting an upward bias on estimated treatment effects for our secondary outcomes). We therefore conclude that it is unlikely that removal of any bias due to selection and missingness in our secondary outcomes would change our conclusions.

**Additional References (not cited in the main manuscript)**

Von Hippel P. (2007), Regression With Missing Ys: An Improved Strategy For Analyzing Multiply Imputed Data. *Sociological Methodology*, 37: 83-117.
